# Gut Microbiome Alterations following Postnatal Iron Supplementation Depend on Iron Form and Persist into Adulthood

**DOI:** 10.3390/nu14030412

**Published:** 2022-01-18

**Authors:** Shasta McMillen, Sydney Thomas, Emily Liang, Eric B. Nonnecke, Carolyn Slupsky, Bo Lönnerdal

**Affiliations:** 1 Department of Nutrition, University of California, Davis, CA 95616, USA; samcmillen@ucdavis.edu (S.M.); emiliang@ucdavis.edu (E.L.); ebnonnecke@ucdavis.edu (E.B.N.); cslupsky@ucdavis.edu (C.S.); 2 Department of Food Science, University of California, Davis, CA 95616, USA; sydthomas@ucdavis.edu

**Keywords:** iron supplement, ferrous sulfate, ferrous bis-glycinate chelate, gut microbiome, metabolomics, rat model, infant nutrition

## Abstract

The gut microbiota is implicated in the adverse developmental outcomes of postnatal iron supplementation. To generate hypotheses on how changes to the gut microbiota by iron adversely affect development, and to determine whether the form of iron influences microbiota outcomes, we characterized gut microbiome and metabolome changes in Sprague-Dawley rat pups given oral supplements of ferrous sulfate (FS), ferrous bis-glycinate chelate (FC), or vehicle control (CON) on postnatal day (PD) 2–14. Iron supplementation reduced microbiome alpha-diversity (*p* < 0.0001) and altered short-chain fatty acids (SCFAs) and trimethylamine (TMA) in a form-dependent manner. To investigate the long-term effects of iron provision in early life, an additional cohort was supplemented with FS, FC, or CON until PD 21 and then weaned onto standard chow. At ~8 weeks of age, young adult (YA) rats that received FS exhibited more diverse microbiomes compared to CON (*p* < 0.05), whereas FC microbiomes were less diverse (*p* < 0.05). Iron provision resulted in 10,000-fold reduced abundance of *Lactobacilli* in pre-weanling and YA animals provided iron in early life (*p* < 0.0001). Our results suggest that in pre-weanling rats, supplemental iron form can generate differential effects on the gut microbiota and microbial metabolism that persist into adulthood.

## 1. Introduction

Iron deficiency (ID)—which affects approximately 10–40% of infants worldwide—can irreversibly disrupt neurodevelopment [1,2,3,4]. Iron supplements and iron-fortified foods are widely used during infancy to prevent ID [5,6]; however, iron provision is not without risks [7,8,9]. Iron provision can have adverse effects in infants, including inflammation [10], growth delay [7,11,12,13], long-term cognitive deficits [14,15,16], and morbidities [11,17,18,19]. The mechanisms underlying these adverse effects are not fully known [8,9,20].

Recent evidence suggests that iron alters development of the gut microbiota in infants [8,19]. The developing microbiota confers an array of health benefits for the infant, such as improved intestinal barrier function and nutrient absorption, as well as infection resistance [21,22,23,24,25]. The development of the microbiota is influenced by the infant’s diet [26,27]. Iron salts, such as ferrous sulfate (FS), are commonly used in supplements and infant formula, despite relatively low bioavailability—only 10% of FS iron is absorbed by infants [28]. Unabsorbed iron in the gut may exert effects on the microbiota [10,19,29,30,31]. Postnatal iron administration has been shown to suppress typical populations comprising the commensal bacteria and promote pathogen-associated bacteria, which may consequently potentiate adverse development outcomes [10,29,30,31,32].

The microbiota can influence their host by producing metabolites that guide nutrient absorption [33], infection resistance [34], and immune regulation [35,36], as well as brain development [37] and behavior [38]. There are few studies on the impact of iron on the metabolism of intestinal microbiota. Furthermore, existing microbiome studies in infants have used different iron forms that may exert differential effects on the microbiota [19].

In the present study, we developed an iron supplementation model in pre-weanling rats to mimic the effects of routine iron administration in healthy, term infants [39]. Rat pups received either FS or ferrous bis-glycinate chelate (Ferrochel^®^; FC), a novel, bioavailable form of iron [40]. Herein, we show that both forms of iron—FS and FC—caused intestinal iron loading and altered the gut microbiome and metabolites in a form-dependent manner.

## 2. Materials and Methods

### 2.1. Animal Experiments

The University of California Institutional Animal Care and Use Committee approved all animal experiments. The postnatal rat iron supplementation model methods have been described in detail previously [39], including rationale for iron dosage. Sprague-Dawley rat litters (*n* = 3 litters per group, culled to 10 pups per litter) were kept with dams and allowed to nurse ad libitum, except for during a brief daily supplementation period. Daily supplementation occurred during PD 2–14 according to random treatment group assignment to vehicle control (CON, 10% sucrose) or 10 mg iron/kg body weight (BW) as ferrous sulfate (FS) or ferrous bis-glycinate chelate (Ferrochel^®^, FC). To determine whether postnatal iron supplementation induced lasting changes to the rat microbiome, additional litters (*n* = 4 litters per group, culled to 10 pups per litter on PD 2) were randomly assigned to FS, FC, or CON groups on PD 2. These litters received daily supplements from PD 2–20 and were weaned on PD 21. All weanling rats were housed with 1–4 littermates and received standard chow (200 mg iron/kg diet as ferrous sulfate; 2018, Teklad Diets, Madison, WI, USA) ad libitum for approximately 6 weeks.

For necropsy of pre-weanling rats, tissues, cecal content, and blood were collected from 4–6 h-fasted animals on postnatal day (PD) 15. For necropsy of adult rats, blood, tissue, and cecal content samples were collected and body, brain, and liver weight were recorded following 4–6 h fasting, on PD 58 ± 4. This age group is referred to as the young adult (YA) group.

### 2.2. Iron Analysis and Hemoglobin

Distal small intestine iron concentration was determined by nitric acid wet-ashing and atomic absorption spectrometry (*n* = 7/group), as previously described [39]. Liver (*n* = 8–10/group) and spleen (*n* = 11–12/group) iron concentrations were also measured in YA rats using this method. For histological evaluation, fresh tissue was perfused with 1× PBS and immersion-fixed in 4% w/v paraformaldehyde (PFA) for 24 h at 4 °C, washed in three changes of 1× PBS, and stored in 70% ethanol at 4 °C. Fixed issues were embedded in paraffin, sectioned, and stained with Perls’ Prussian blue for iron detection (*n* = 6/group, 3 litters/group) at the UC Davis School of Veterinary Medicine Anatomic Pathology Laboratory. Hemoglobin was measured in fresh, whole blood collected from YA rats using a kit (Cat#MAK115-1KT, Sigma-Aldrich, St. Louis, MO, USA).

### 2.3. Distal Small Instestine Morphology

Following PFA-fixation and paraffin embedding, as described above, two 1 cm distal sections of intestine from each pup (*n* = 6/group, 3 litters/group) were stained with H&E by UC Davis School of Veterinary Medicine Anatomic Pathology Laboratory for morphological evaluation. Microscope images (20× objective lens) were analyzed using ImageJ software (v1.51; [41]) to quantify villus height and crypt depth. All images were obtained in a blinded fashion. Data represent means of 20 villi and 20 neighboring crypts for each biological replicate.

### 2.4. Distal Small Intestine Gene Expression

Approximately 1 cm distal intestine was collected during necropsy, perfused with 1× PBS, and stored in RNA*later*^®^ (Thermo Fisher Scientific ^TM^, Waltham, MA, USA) for 24 h at room temperature, and then at −20 °C until RNA extraction. RNA was extracted from intestine using TRIzol (Invitrogen ^TM^, Carlsbad, CA, USA) according to the manufacturer’s instructions. RNA was reverse transcribed using the High-Capacity cDNA Archive Kit (Applied Biosystems ™, Cat#4374966, Applied Biosystems ^TM^, Foster City, CA, USA), according to the manufacturer’s instructions. Real-Time quantitative PCR was performed using a Bio-Rad CFX96 Real-Time machine with iTaq Universal SYBR Green Supermix (Cat#1725121, Bio-Rad, Hercules, CA, USA). Fold-change in mRNA gene expression was calculated using the 2^∆∆*Ct*^, with *Actb* serving as the housekeeping gene. Primer sequences are listed in Table S1. Intron-spanning primers were designed using MacVector v18.0.1 (74) software (MacVector Inc., Apex, NC, USA).

### 2.5. Cecal DNA & Metabolite Extraction

Cecal content was collected in sterile tubes and flash frozen in liquid nitrogen and stored at −80 °C. DNA and metabolites were extracted from the same samples, under the same thaw cycle. All surfaces and equipment used during extraction were cleaned with 70% ethanol and decontaminated with DNA Away (Cat#2123628, Thermo Fisher Scientific ^TM^, Waltham, MA, USA). Cecal content was thawed on ice, weighed, and homogenized in 1.5 mL of sterile, ice cold 1× PBS. Samples rested on ice at intervals during homogenization. Following homogenization, samples were pelleted by centrifugation at 14k rcf. DNA was extracted from the pellet, and metabolites were isolated from the supernatant. Each sample of supernatant was filtered through a 0.22 um syringe and then loaded onto a pre-washed 3 kDa Amicon Ultra Centrifugal Filter Unit (Cat# UFC500396, Millipore Sigma, Burlington, MA, USA) to remove contaminants prior to storage at −80 °C. DNA was extracted from the sample pellet using the DNeasy PowerLyzer PowerSoil Kit (QIAGEN, Hilden, Germany) according to manufacturer’s instructions, with the following modifications: sample pellets were vortexed with beads and bead solution and incubated for 10 min at 65 °C, followed by 10 min at 95 °C prior to bead beating for 2 min at 6.5 m/s to ensure complete lysis of bacterial cells. Extracted DNA was stored at −80 °C.

### 2.6. 16S rRNA Amplicon Library Construction, Sequencing, and Processing

Barcoded primers with Illumina sequencing adapters (Table S1) were combined with GoTaq^®^ Master Mix for PCR (Promega Corporation, Madison, WI, USA) to amplify the variable region 4 (V4) of the prokaryotic 16S rRNA gene in the cecal DNA samples (primer sequences in Table S1). Libraries were constructed from PCR products that had been pre-checked for quality on an agarose gel with SYBR ™ Safe (Thermo Fisher Scientific ^TM^, Waltham, MA, USA) and purified using a kit (QIAGEN, Hilden, Germany). Libraries were sequenced with an Illumina MiSeq platform at the DNA Technologies and Expression Analysis Core Laboratory at UC Davis.

A total of 17 million paired-end Illumina reads passed initial filtering with an overall Q30 of >80%. Reads were imported into QIIME 2, v2020.2 [42] and demultiplexed using the emp-paired plugin [43,44]. Demultiplexed reads were filtered and denoised with the DADA2 plugin to identify unique 16S amplicon sequence variants (ASVs) [45]. To optimize read quality, forward and reverse sequences were trimmed to 240 bp and 200 bp, respectively, and reads were filtered to allow a maximum of two expected errors. In the PD 15 age group (*n* = 27–29 per treatment group), a total of 3.3 million reads passed quality filtering and were merged, and 1111 ASVs were identified; in the YA age group (*n* = 22–23 per treatment), 4.0 million reads passed quality filtering and were merged, and 1889 ASV’s were identified. Features (ASV’s) and their representative sequences were applied to the phylogeny align-to-tree-mafft-fasttree pipeline to construct a phylogenetic tree. Rarefaction plotting and computation of alpha-diversity indices were performed using the alpha-rarefaction plugin. ASV’s were assigned to taxonomy using the feature-classifier plugin [46] with a Bayes classifier that was pretrained on a Silva 138 V4 reference database [47,48,49,50]. Finally, the ASV table, representative sequence table, and constructed phylogenetic tree were imported into R (v4.1.0) using the qiime2R package for beta diversity, relative abundance, and statistical analysis, described in further detail below.

### 2.7. NMR Metabolomics

Cecal filtrate was mixed with EDTA/K_2_HPO_4_ buffer (pH 8.07) such that the final concentrations were 2 mM/10 mM, respectively. To this cecal mixture (CM), an internal standard (IS) containing 5 mM 3-trimethylsilyl-1-propanesulfonic acid-d6 (DSS-d6), in 99% deuterium oxide (D_2_O) and 0.2% sodium azide (NaN_3_) (Chenomx; AB, Canada) was added in a 1:10 IS:CM ratio. Sample pH was adjusted to between 6.95 and 7.10 using HCl or NaOH, and samples were then placed into 3 mm NMR tubes and maintained at 4 °C prior to acquisition. NMR spectra were recorded on a 600 MHz AVANCE system (Bruker, Billerica, MA, USA) equipped with a SampleJet using the noesypr1D pulse sequence at 25 °C, as described in [51]. The corresponding spectra were analyzed using Chenomx NMRSuite Professional v.8.5 [52], and output as concentrations.

### 2.8. Statistical Analysis

Statistical analysis and plotting were performed using GraphPad Prism (v8), and R (v4.1.0) with the following core packages: ggplot2 (v3.3.3), phyloseq (v1.36.0), microbiome (v1.14.0), qiime2R (v0.99.6), car (v3.0-10), dunn.test (v1.3.5), pairwiseAdonis (v0.0.1), and DESeq2 (v1.32.0). *p*-values < 0.05 were considered significant.

Differences in iron concentration were detected among treatments with Kruskal–Wallis and Dunn’s tests. Differences in hemoglobin, intestinal morphology, and gene expression among treatment groups were detected with one-way ANOVA and Tukey’s tests. Differences in body, brain, and liver weight among groups in male and female YA rats were also detected with one-way ANOVA and Tukey’s tests.

For the 16S rRNA amplicon sequencing library analysis, a total of 153 samples were sequenced from both age groups of the original 162 (90 pups and 72 YA rats) planned for collection. Two CON cecal samples were not collected during necropsy in error, and four samples (three from FS and one from FC) yielded poor quality DNA due to a small cecal sample size, leaving 84 pup samples to be sequenced (*n* = 27–29/group, 3 litters/group). Three YA rats were not sequenced: one CON was not collected at necropsy in error, and one each from FS and FC were lost in error during DNA extraction, leaving 69 YA samples to be sequenced (*n* = 23/group, 3 litters each group). Following sequencing, four additional samples were excluded from microbiome analyses due to zero reads following filtering and denoising. These samples were from YA rats in the FC group, and for unknown reasons produced only poor-quality reads during sequencing that were then filtered out.

Alpha-diversity was assessed by rarefying at depths from 1–10,000 sequences. Three different metrics of alpha-diversity were estimated at each sampling depth: Faith’s Phylogenetic Diversity Index, Shannon’s H Index, and richness (amplicon sequence variant, ASV, count). For each alpha-diversity metric, sample values were calculated by taking the mean of 10 iterations at that sampling depth. Significant differences in each alpha-diversity metric due to treatment and among treatment groups were detected at each depth by Kruskal–Wallis test, and Dunn’s post-hoc test, respectively. Beta-diversity was assessed by log-transforming ASV counts and by using principal coordinate analyses (PCoA’s) to evaluate three beta-diversity metrics per age group: Weighted UniFrac, Unweighted UniFrac, and Bray–Curtis. In each age group and for each beta-diversity metric, PERMANOVA’s were used detect the effects of treatment, sex, litter, and variable interactions on community heterogeneity. For each PERMANOVA, 10,000 randomizations were performed to test for significance. For differential phylum and genus abundance, ASV counts were aggregated to Phylum or Genus level, respectively, and pseudo-counts of 1 were added to all ASV’s to remove 0 values. Differential abundance due to treatment was assessed at both taxonomic ranks for both ages using DESeq2 [53,54]. Pairwise results were extracted and plotted as log_2_[Fold Change], and FDR-adjusted *p*-values < 0.05 were considered to be a significantly different change between groups.

One cecal metabolite extract sample belonging to the pup CON group was removed due to excess zeros and lack of confidence in quantification using Chenomx (see metabolite methods above). Metabolite concentrations were converted to nmol/g wet weight by multiplying the concentration by the volume of buffer each was extracted into and dividing by the wet weight of the cecal content before extraction. Sex and litter effects were assessed separately using the multivariate approach of PCoA followed by PLS-DA; both methods demonstrated uninfluential overlap within metabolome. Metabolites were analyzed using Kruskal–Wallis followed by Dunn’s multiple comparison test in Prism GraphPad v 6.0c.

## 3. Results

### 3.1. Effects of Postnatal Iron Supplementation on the Distal Small Intestine in Pups

#### 3.1.1. Distal Small Intestine Iron Loading

**Figure 1:**
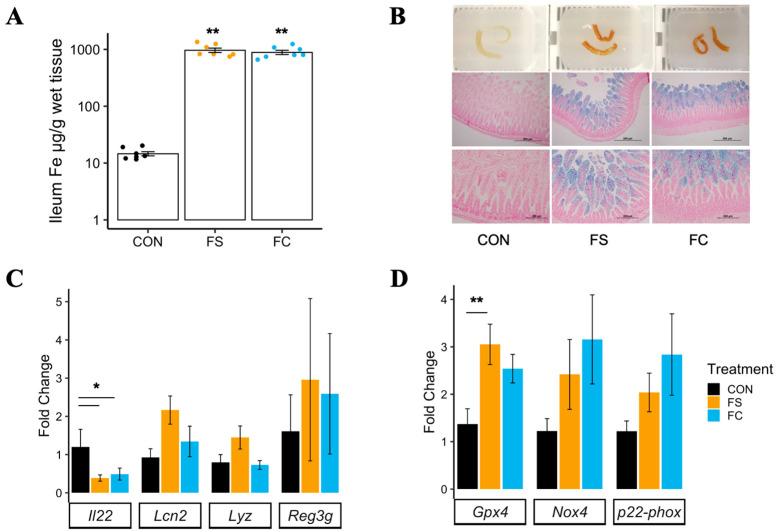
Postnatal iron supplementation with ferrous sulfate (FS) or ferrous bis-glycinate (Ferrochel^®^; FC) leads to iron loading in the distal intestine and altered intestinal gene expression in pre-weanling rats. (**A**) Iron concentration in distal small intestine tissue (*n* = 7/group, 2 litters/group) collected from rat pups at PD 15 following 13 days of daily iron supplementation with FS or FC, or vehicle control (CON), assessed by atomic absorption spectrometry. Biological replicates are shown as individual data points with mean ± SEM. Differences in iron concentration among groups were detected with a Kruskal–Wallis test and Wilcox pairwise test. (**B**) Top row: distal small intestine samples exhibiting darkening effect of iron supplementation. Middle and bottom row: 10× (scale = 500 µm) and 20× (scale = 200 µm) objective microscope images of distal small intestine sections stained for iron with Perls’ Prussian blue staining (representative samples; *n* = 6/group, 3 litters/group). (**C**) mRNA expression of genes involved in regulation of mucosal immunity, quantified by real-time PCR (*n* = 5–8/group, 2 litters/group). (**D**) mRNA expression of genes involved in regulation of ferroptosis, an iron overload-dependent form of cell death. Gene expression values are shown as mean fold-change ± SEM. mRNA expression was normalized to *Actb*, and differences were detected among groups by one-way ANOVA and Tukey’s multiple comparisons. *p*-value summary: *, *p* < 0.05; **, *p* < 0.01.

Iron concentration was measured in the distal small intestine of ferrous sulfate (FS) iron, ferrous bis-glycinate chelate (FC) iron, and vehicle control-supplemented (CON) pups, where tissue darkening was observed in samples collected from iron-supplemented pups (Figure 1B; top row). Both FS and FC treatments increased tissue iron concentration in the distal intestine (*p* < 0.01; Figure 1A), an effect independent of iron form (*p* = 0.26). Perls’ Prussian blue staining for iron in fixed distal small intestine sections confirmed mucosal iron loading (Figure 1B; middle and bottom rows).

#### 3.1.2. Distal Small Intestine Morphology

Previous studies have reported cytotoxic effects of iron loading in the gastrointestinal mucosa, leading to tissue necrosis and atrophy of intestinal villi [55,56]. Despite marked iron loading in the distal small intestine with iron treatment, no alterations were observed in villus height, crypt depth, or villus height/crypt depth ratio (Figure S1).

#### 3.1.3. Distal Small Intestine Gene Expression

In adult animals, interleukin 22 (Il22) is constitutively expressed by intestinal immune cells and regulates the mucosal barrier and intestinal inflammation. Pro-inflammatory signaling upregulates Il22, which then signals epithelial cell survival and proliferation to promote mucosal barrier integrity [57]. Il22 also promotes intestinal antimicrobial activity. Anti-microbial genes *Lcn2*, *Reg3g*, and *Lyz* are modulated by Il22 signaling [58,59,60,61]. A decrease in II22 mRNA expression in the distal small intestine was found for both iron treatment groups (*p* < 0.01; Figure 1C). No differences in expression of *Lcn2*, *Lyz*, or *Reg3g* were found among treatment groups.

Ferroptosis is an iron-dependent form of cell death that may be initiated in iron-loaded cells [62]. It is currently unclear if iron loading alone can increase ferroptotic activity in the various cells comprising the small intestine [63]. *Gpx4* expression was elevated in the FS group but not in the FC group compared to CON (*p* < 0.01; Figure 1D). Increased *Gpx4* expression in FS-treated samples is suggestive of an antioxidant, anti-ferroptotic response. *Gpx4* enzymatic activity mitigates damage by lipid peroxidation and is suppressed during ferroptosis [64]. No effect of treatment was observed in *Nox4* or *p22-phox*, genes involved in NADPH oxidase, suggesting ferroptosis was not induced due to iron loading [62,65].

### 3.2. Effect of FS vs. FC on Cecal Microbiome in Pups

#### 3.2.1. Cecal Microbiome Diversity

**Figure 2:**
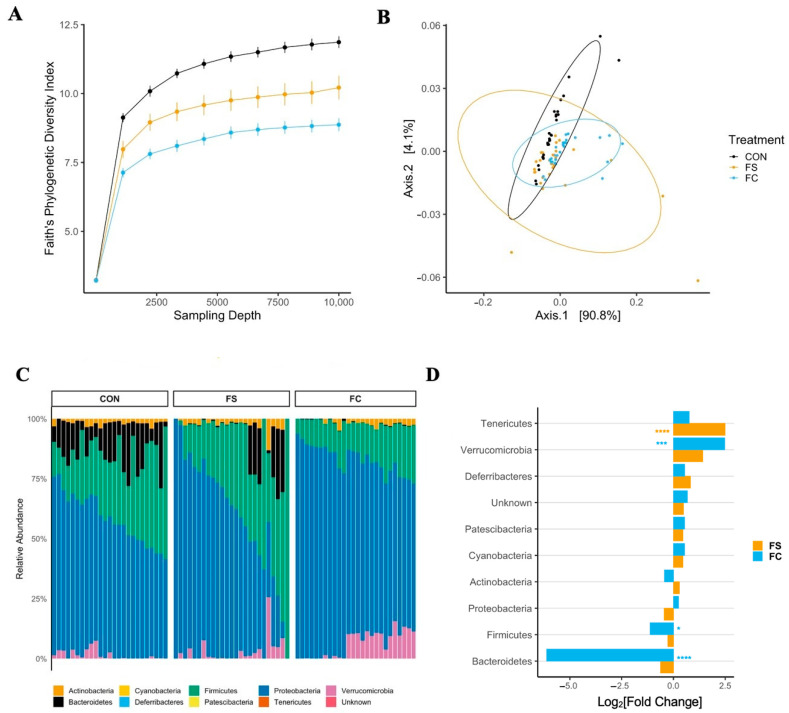
Alterations in the cecal microbiome depend on iron form. (**A**) Alpha-diversity rarefaction plot depicting Faith’s Phylogenetic Diversity (FPD) Index by treatment group and sampling depth, shown as mean ± SEM (*n* = 20–29/(group × sampling depth), 3 litters/group). Iron treatments decreased FPD; this was true for all sampling depths >1, with the largest effect upon FC treatment (*p* < 0.05). (**B**) Principal coordinate axes 1 and 2 from Principal Coordinate Analysis (PCoA) of Weighted UniFrac distances in pups (*n* = 27–29/group, 3 litters/group) following FS or FC supplementation; no separation due to treatment group detected (*p* = 0.08). (**C**) Relative abundance of phyla (%) by treatment group; each column represents an individual pup (*n* = 25–26/group, 3 litters/group). (**D**) Differential phyla abundance, represented as log_2_[Fold Change] from CON for each iron treatment group (*n* = 27–29/group, 3 litters/group); phyla are ordered by magnitude of change and bars are labeled with color according to iron treatment group. Repeated Kruskal–Wallis tests with Dunn’s multiple comparisons were used to test for differences in FPD among groups at each sampling depth. A PERMANOVA test was applied to detect microbiome compositional dissimilarity among treatment groups, using a nested model; litter was nested within treatment. Additional alpha- and beta-diversity metrics from this age group are included in Figure S2, and *p*-values for diversity analyses in pups are listed in Table S2. Differential abundance of phyla was assessed with DESeq2, and FDR-adjusted *p*-values <0.05 from pairwise comparisons were considered significant. *p*-values from the pairwise phyla differential abundance results are provided in Table S3. *p*-value summary: *, *p* < 0.05; **, *p* < 0.01; ***, *p* < 0.001; ****, *p* < 0.0001.

Iron supplementation reduced alpha diversity at all sampling depths >1 in all metrics (*p* < 0.0001; Figure 2 and Figure S2); this effect was greater in the FC group (FC vs. CON, *p* < 0.001; FC vs. FS, *p* < 0.05). Faith’s Phylogenetic Diversity (FPD, Figure 2A) and ASV count (species richness, Figure S2) diversity were lower in the FS group compared to CON (*p* < 0.01), but Shannon’s H Index, an alpha-diversity metric that assesses both richness and evenness, was similar between FS and CON (Figure S2).

#### 3.2.2. Cecal Microbiome Composition

Cecal bacterial community dissimilarity (beta-diversity) effects were observed due to treatment and litter, but no effect of sex on beta-diversity was observed across metrics. There were effects of treatment on Bray–Curtis and unweighted UniFrac distances (Figure S2; *p* < 0.0001) as well as treatment:litter interaction (*p* < 0.0001). The effects of treatment:sex and treatment:sex:litter interactions on unweighted UniFrac distance were also found (*p* < 0.01). Pairwise analysis of Bray–Curtis and unweighted UniFrac results revealed separation of iron treatment groups from CON (*p* < 0.01) and separation between iron groups (*p* < 0.01). No effect of treatment was found on weighted UniFrac distance (Figure 2B; *p* = 0.08). These and the alpha-diversity results indicate that the overall gut microbiome community was altered by iron treatment, while specific effects depended upon litter (i.e., baseline microbiome). The microbiome shifts observed with this early iron treatment support the idea that exogenous iron may be disruptive to early microbiome colonization. *p*-values from alpha- and beta-diversity analyses in pups are listed in Table S2.

#### 3.2.3. Cecal Microbiome Differential Abundance

In general, relative phyla abundance was comparable to what has been reported previously at this developmental stage [66]. Dominant phyla included Proteobacteria and Firmicutes, as well as Bacteroidetes, Actinobacteria, and Verrucomicrobia (Figure 2C). The relative abundance of bacterial phyla was altered with iron supplementation according to iron form (Figure 2C,D and Figure S3). In FC-treated pups we found a 100-fold lower abundance of Bacteroidetes (*p* < 0.0001) and lower Firmicutes (*p* < 0.05) compared to CON; Verrucomicrobia were over 6-fold elevated (*p* < 0.001). The relative abundances of Bacteroidetes, Firmicutes, and Verrucomicrobia were similar between FS and CON. Conversely, Tenericutes abundance was elevated with FS treatment compared to CON (*p* < 0.0001), while it was similar between FC and CON.

Loss of Bacteroidetes and Firmicutes with FC treatment suggests potential adverse effects on health with this form of iron. A high-fat diet or high-fat, high-sugar diet in mice can lower Bacteroidetes abundance, and this is associated with metabolic dysfunction. Similarly, low fiber intake also results in decreased Bacteroidetes abundance in mice. The lower abundance of Firmicutes in FC is unlikely to be beneficial to the host, as Firmicutes generate SCFA and other metabolites that may be beneficial to the host [67]. The statistical results of phylum differential abundance in pups are listed in Table S3.

**Figure 3:**
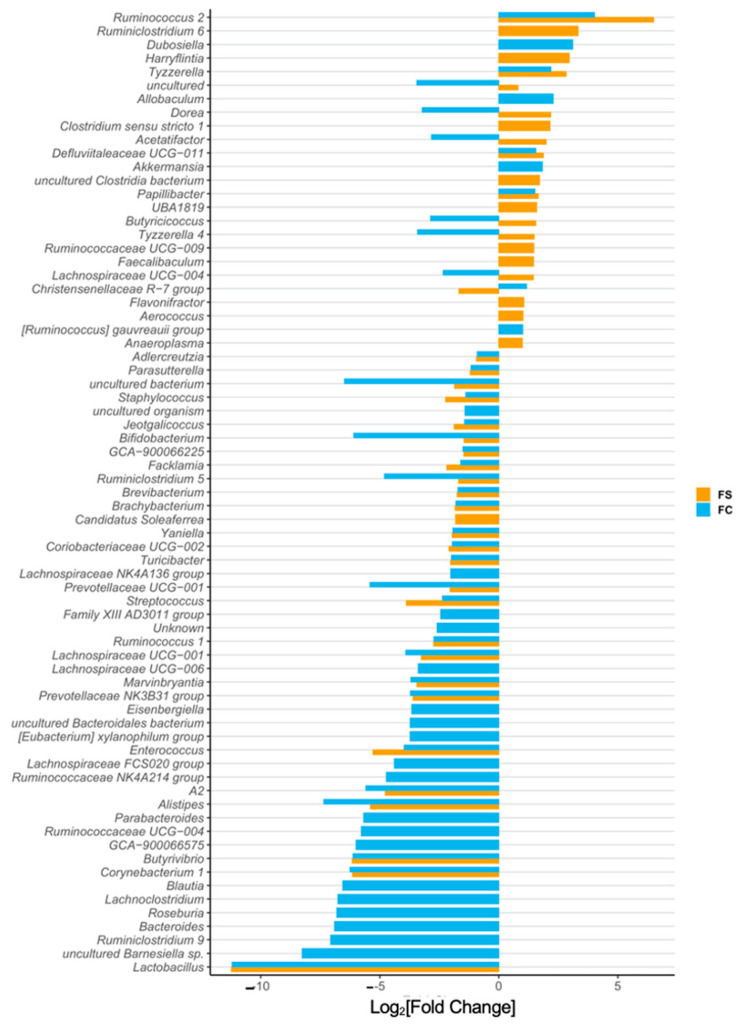
Differentially abundant genera due to iron form (*n* = 27–29/group, 3 litters/group). Differentially abundant genera are represented as log_2_[Fold Change] from CON for each iron group. Genera are ordered by magnitude of change. Differential abundance was assessed with DESeq2, and FDR-adjusted *p*-values < 0.05 were considered significant. All significant results are shown in the plot, and their adjusted *p*-values are listed in Table S4.

We identified 73 differentially abundant genera among CON, FS, and FC pups (Figure 3 and Figure S3). Iron-treated pups had a 10,000-fold lower abundance of *Lactobacilli* compared to CON pups (*p* < 0.0001); this effect was the largest we observed in all the 73 differentially abundant genera. *Bifidobacteria* were also less abundant in iron-treated pups (*p* < 0.05), as were *Turicibacter* (*p* < 0.0001), while *Ruminococcus 2* relative abundance was elevated (*p* < 0.0001). *Bacteroides* and *Parabacteroides* were 100-fold less abundant (*p* < 0.0001) in FC compared to CON, but these were uninfluenced by FS treatment. No change in *Escherichia-Shigella* abundance was observed among treatment groups. The abundance of *Clostridium sensu stricto* 1 was increased in FS compared to CON (*p* < 0.0001) but was comparable between CON and FC.

*Lactobacillus* and *Bifidobacterium* are considered beneficial, so their reduced abundance with iron treatment could imply adverse health and development effects [19,21,67]. These results also imply that FC changes are potentially less adverse than FS-induced microbiome changes, because *Bacteroides* is reduced with FC and *Clostridium* is increased with FS. *Bacteroides* are associated with metabolic dysfunction and *Clostridium* are associated with more pathogenic activity [67]. The statistical results of genus differential abundance in pups are listed in Table S4.

### 3.3. Effects of FS or FC on Cecal Metabolites in Pups

**Figure 4:**
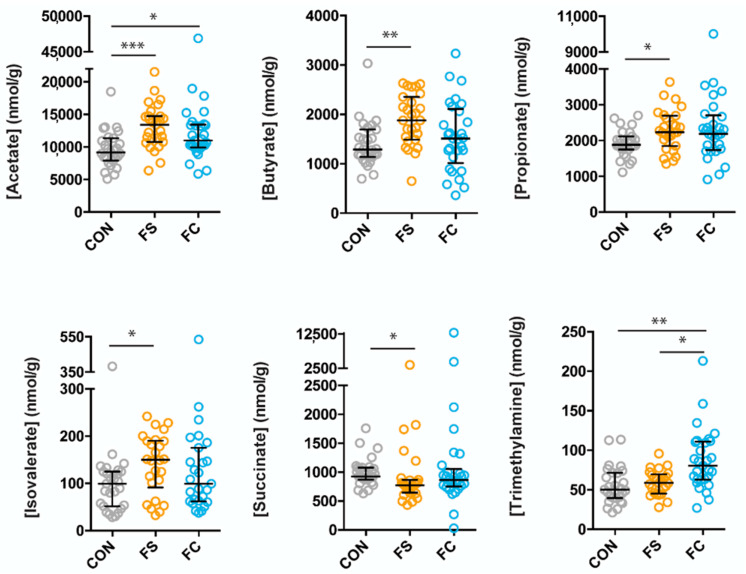
Cecal metabolite differences due to iron form. Metabolites were analyzed by Kruskal–Wallis with Dunn’s multiple comparisons. All metabolites shown are significant by Kruskal–Wallis. Median, and interquartile range of concentrations are indicated (*n* = 27–29/group, 3 litters/group). *p*-value summary: *, *p* < 0.05; **, *p* < 0.01; ***, *p* < 0.001.

A total of 25 metabolites were identified and quantified from rat pup cecal extracts. These metabolites included short-chain fatty acids (SCFA): acetate, butyrate, propionate, valerate, and isovalerate; amino acids: alanine, arginine, glutamate, glycine, isoleucine, leucine, methionine, proline, and valine; organic acids: 2-oxoglutarate, 3-hydroxybutyrate, 4-aminobutyrate, 5-aminopentanoate, formate, lactate, pyruvate, succinate; as well as methanol, trimethylamine (TMA), and sialic acid (Figure 4 and Figure S4). Metabolites that differed between the groups were: acetate (*p* = 0.0002), butyrate (*p* = 0.002), propionate (*p* = 0.03), isovalerate (*p* = 0.03), succinate (*p* = 0.01), and TMA (*p* = 0.003). Interestingly, differences in metabolite concentrations were largely due to FS treatment, including acetate (higher in FS, *p* = 0.0001), butyrate (higher in FS, *p* = 0.002), propionate (higher in FS, *p* = 0.04), isovalerate (higher in FS, *p* = 0.02), and succinate (lower in FS, *p* = 0.01)). Only two metabolite concentrations differed between CON and FC treatment groups: acetate (higher in FC, *p* = 0.03) and TMA (higher in FC, *p* = 0.003) (Figure 4). Comparison of the concentrations of the remaining 19 metabolites is shown in Figure S4.

### 3.4. Long-Term Effects of FS vs. FC in Young Adult Rats

#### 3.4.1. Body, Brain, and Liver Weight, and Iron Status

We measured body, liver, and brain weight in YA rats (*n* = 22–23/group). We also measured hemoglobin, liver iron concentration, and spleen iron to assess iron status. Male and female results were separated, then tested for treatment effects. Iron treatment did not affect these parameters (Table S5).

#### 3.4.2. Cecal Microbiome Diversity

**Figure 5:**
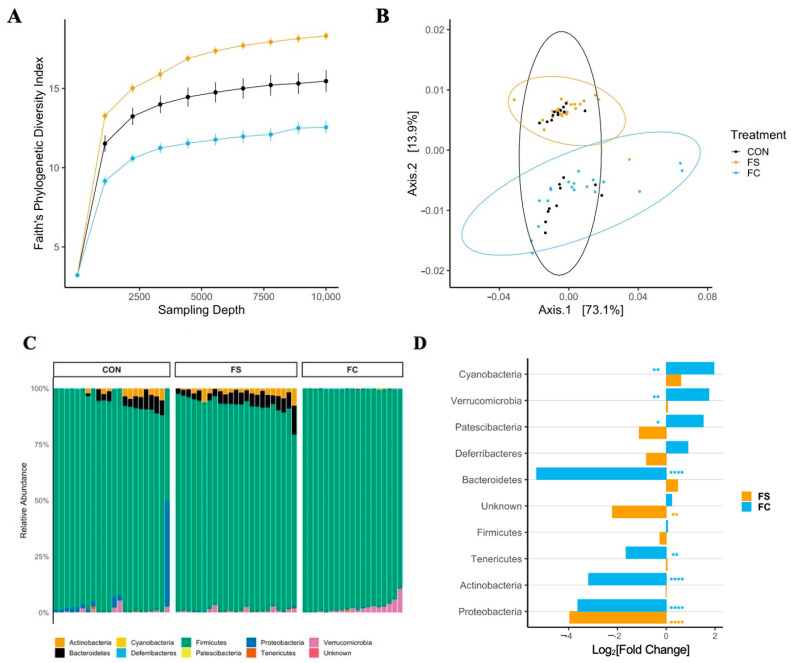
Cecal microbiome effects in young adult (YA) rats supplemented with FS or FC during the pre-weaning period. (**A**) Alpha-diversity rarefaction plot depicting Faith’s Phylogenetic Diversity (FPD) Index by treatment group and sampling depth, shown as mean ± SEM (*n* = 12–23/(group *×* sampling depth), 4 litters/group). Postnatal FS treatment increased YA rat cecal microbiome FPD, while FC decreased FPD compared to CON across all sampling depths >1 (*p* < 0.05). (**B**) Principal Coordinate Analysis (PCoA) of Weighted UniFrac distances in YA rats (*n* = 19–23/group, 4 litters/group), depicting separation due to treatment group (*p* < 0.01) (**C**) Relative abundance of phyla (%) by treatment group; each column represents an individual animal (*n* = 19–23/group, 4 litters/group). (**D**) Differential phyla abundance, represented as log_2_[Fold Change] from CON for each iron group (*n* = 19–23/group, 4 litters/group); phyla are ordered by magnitude of change and bars are labeled with color according to iron group. Repeated Kruskal–Wallis tests with Dunn’s multiple comparisons were used to test for differences in FPD among groups at each sampling depth. Weighted UniFrac PCoA results were applied to a PERMANOVA test. Additional alpha- and beta-diversity metrics for YA rats are included in Figure S2. *p*-values for YA diversity analyses are listed in Table S6. Differential abundance of phyla was assessed with DESeq2, and FDR-adjusted *p*-values <0.05 from pairwise comparisons were considered significant. *p*-values from the pairwise phyla differential abundance results are provided in Table S6. *p*-value summary: *, *p* < 0.05; **, *p* < 0.01; ***, *p* < 0.001; ****, *p* < 0.0001.

Postnatal supplementation with both iron forms modified alpha-diversity in YA rats with all metrics at all sampling depths >1 (*p* < 0.0001; Figure 5A and Figure S4A,C). The direction of effect depended on iron form; FS increased diversity compared to CON (*p* < 0.05), while FC decreased diversity (*p* < 0.05).

#### 3.4.3. Cecal Microbiome Composition

In YA rats, we observed an effect of treatment on bacterial community dissimilarity with all three metrics (*p* < 0.01; Figure 5B and Figure S4B,D). The effects of treatment:litter interaction on Bray–Curtis and unweighted UniFrac distances were also observed (*p* < 0.0001; Figure S5B,D). Pairwise analysis of treatment groups revealed separation of iron-treated groups from CON and separation between iron groups (*p* < 0.05), indicating that postnatal iron supplementation and iron form affected YA cecal bacterial community composition. These and the alpha-diversity results indicate differential effects on the overall microbiome community with FS vs. FC iron treatment in early life. Indeed, FS- and FC-treated microbiomes appeared more distinct from each other than from CON. This suggests the different forms of iron used for supplementation in early life may initiate divergent adult gut microbiome phenotypes. *p*-values from alpha- and beta-diversity analyses in YA rats are listed in Table S6.

#### 3.4.4. Cecal Microbiome Differential Abundance

A lower abundance (~10-fold) of Proteobacteria was found in YA rats that were treated with FS and FC (*p* < 0.0001; Figure 5D). Consistent with the results in pups, FC-treated YA rats had less Bacteroidetes than CON (*p* < 0.0001) and elevated Verrucomicrobia (*p* < 0.01); however, parallel changes did not occur in FS-treated rats. In YA rats, but not pups, Patescibacteria (*p* < 0.05) and Cyanobacteria (*p* < 0.01) increased in abundance in FC compared to CON, and Actinobacteria was decreased (*p* < 0.0001), while these were unchanged in the FS group. Unidentified organisms (*p* < 0.01) were decreased with FS compared to CON but were unaffected by FC. As in pups, the reduced abundance of Bacteroidetes due to FC treatment in early life may suggest adverse effects to health and metabolism [67]. However, the reduced Proteobacteria abundance with iron treatment may have beneficial effects, because this phylum contains many pathogenic gram-negative bacteria, and elevated abundance may cause dysbiosis [68]. The statistical results of phylum differential abundance in YA rats are listed in Table S7.

**Figure 6:**
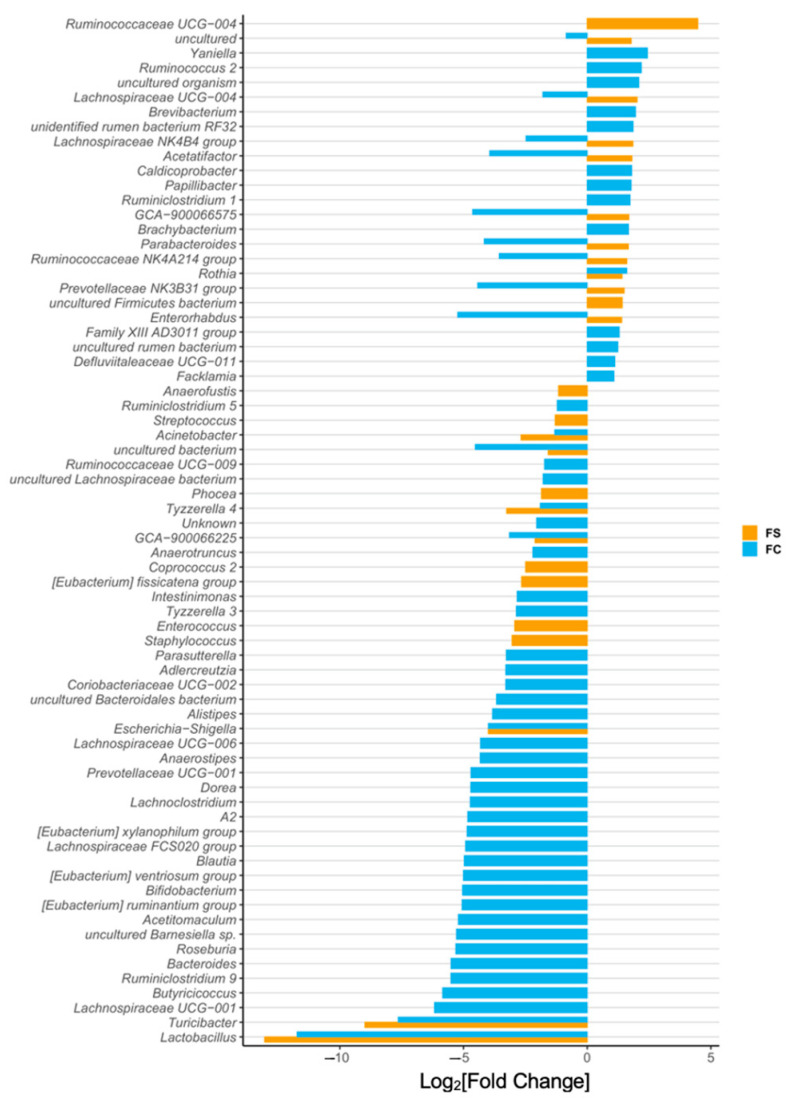
Differentially abundant genera in young adult (YA) rats due to iron form (*n* = 19–23/group, 4 litters/group). Differentially abundant genera are represented as log_2_[Fold Change] from CON for each iron group. Genera are ordered by magnitude of change. Differential abundance was assessed with DESeq2, and FDR-adjusted *p*-values < 0.05 were considered significant. All significant comparison results are shown in the plot, and their adjusted *p*-values are listed in Table S8.

We identified 95 differentially abundant genera among YA rat treatment groups (Figure 6 and Figure S6). Consistent with results in pups, iron-treated rats had more than 10,000-fold lower abundance of *Lactobacillus* compared to CON rats (*p* < 0.0001)—the largest effect observed in all 95 differentially abundant genera in YA rats. *Turicibacter* abundance was 1000-fold lower in iron-treated rats (*p* < 0.0001). In iron-treated YA rats, but not in pups, abundance of *Escherichia-shigella* was around 10-fold lower than CON (*p* < 0.0001), and this effect was similar between FS and FC. Also consistent with pups, *Bifidobacterium* abundance in FC rats was 100-fold lower than in CON (*p* < 0.0001), although it was similar between FS and CON; *Bacteroides* abundance was 100-fold lower in FC rats compared to CON (*p* < 0.0001) and was comparable between FS and CON. The reduction of *Escherichia-shigella* bacteria with early-life iron treatment is suggestive of beneficial effects, but the reduction of *Lactobacillus* is inconsistent with that notion. Reduced *Bacteroides* with the FC treatment may be beneficial, but reduced *Bifidobacterium* is unlikely to be beneficial to health [67]. The statistical results of genus differential abundance in YA rats are listed in Table S8.

## 4. Discussion

Current research suggests that supplemental iron causes unfavorable changes to the infant gut microbiota [8,19], which may explain how iron can adversely affect infant health [11,17,69], growth [11,12], and development [14,15,70,71,72]. Few studies have examined the effects of iron on the microbiota of healthy, term infants, despite evidence that iron-replete infants may experience more adverse effects of exogenous iron [11,12,14,17,69,73]. It has been unclear if iron effects on the microbiota persist beyond weaning, or whether the iron form influences such outcomes. We gave pre-weanling Sprague-Dawley rats either FS or FC iron (10 mg iron/kg body weight per day; representative of formula iron intake), or a vehicle control (CON) to model the outcomes of iron supplementation in healthy infants [39]. Iron treatment altered the pre-weanling microbiome and its associated metabolites, and long-term microbiome effects in adult animals were also observed. Microbiome alterations depended upon the form of iron—FS or FC. These findings will generate hypotheses regarding how iron can disrupt health and development in infants.

A recent systematic review concluded that iron supplementation can cause diarrhea in infants—the authors indicated this phenotype may be a direct effect of iron toxicity on host cells or alternatively due to deleterious alterations in the microbiota [18]. To our knowledge, we are the first to report outcomes of iron supplementation in early life on distal small intestine morphology (Figure S1) and ferroptosis (Figure 1D), where iron was provided at physiologically appropriate levels. We did not observe signs of intestinal necrosis or mucosal atrophy in iron-treated pups, despite marked iron loading (Figure 1A,B). This iron loading, combined with the absence of mucosal atrophy, may suggest that the distal small intestine exhibits additional resistance to iron excess. Because we did not report on diarrhea or intestinal iron absorption activity, more experiments are required to understand the role of distal small intestine iron loading on the health and morbidity outcomes of iron supplementation. Our results suggest that, in specific pathogen free rats, iron supplementation at physiological levels does not induce direct cytotoxic effects on the intestine. It is therefore likely that alterations in the gut microbiota may be mediating adverse gastrointestinal outcomes of iron. Future work may assess how baseline microbiome and pathogen burden impact the risk of diarrhea following iron supplementation.

In our study (Figure 2), iron supplementation was found to alter the pre-weanling gut microbiome, including significant heterogeneity of the microbiome communities (beta-diversity) and significantly lower species richness (alpha-diversity). Additionally, microbiome effects were found in adult rats that received iron from PD 2 up to weaning (Figure 5), despite adherence to normal rat chow for ~six weeks prior to sample collection —a timeframe spanning the sexual maturation of the animal. Adult animals treated with FC prior to weaning had microbiomes that were distinct from FS-treated animals. Our results confirm previous reports that iron supplementation alters the gut microbiota [10,30,31,74]. The findings in adult animals suggest lasting effects of early-life iron supplementation; however, a longitudinal study is necessary to determine the influence of age and weaning on the persistence of microbiome alterations from early-life iron provision. The changes in bacterial abundance at the phylum and genus levels at both ages imply potential adverse effects to health [67]. The differential effects of FS and FC on gut bacterial abundance align with recent evidence that the form of iron is relevant when considering health outcomes of iron provision [75,76].

The gut microbiota can be easily disturbed in early life before the community stabilizes into an adult-like composition, at around 2–3 years of age [22,24,77]. Breastfeeding provides key probiotics and prebiotics for the developing gut, and a growing number of studies suggest long-term health disparities caused by formula-feeding may be attributed to the disruption of early gut microbiota [21,23,78,79]. The typical infant formula provides approximately twenty-fold more iron than breast milk [80]. Early introduction of formula or weaning foods—often fortified with FS iron—disrupts the natural temporal development of the gut microbiota, which has negative implications for infant health and development [23,77,78]. Host-microbe interactions between humans (or other mammals) and milk-associated microbes *Bifidobacterium* or *Lactobacillus* typically protect health and guide development [27,81,82,83]. *Bifidobacteria* predominate in the breast-fed infant gut, gradually subsiding as gut communities become more diverse upon introduction of complementary foods [21,22,27,77]. *Lactobacilli* predominance in nursing rat pups is thought to be symbiotic and supported by rat milk [82,84,85], and much like *Bifidobacteria* in humans, relative abundance is reduced at weaning [84]. Although various effects of iron provision were observed, the greatest effect was a reduction of *Lactobacilli* in both pre-weanling and YA rats (Figure 3 and Figure 6). Moreover, we also observed lower *Bifidobacterium* and enriched *Ruminococcus* genera due to iron. These results reflect previous microbiome outcomes in controlled iron studies [10,29,31,74], as well as the effects of formula on the gut microbiota [51,79]. It remains plausible that iron supplementation causes adverse health effects in infants by precluding symbiotic, milk-associated microbes in the developing gut. Considering the difference in iron content between iron-fortified formula [80] and human [84,86] or rat milk [87] and the similar effects of iron and iron-fortified formula on the microbiome [19,79], future studies should define the role of iron in formula-induced changes in the gut microbiota.

Various constituents of foodstuffs in the gut lumen are metabolized by the microbiota, forming a complex metabolic network that contributes to the metabolism of the host. Microbial-derived metabolites are essential for proper development of immunity and cognitive functions, as well as for healthy metabolic function [34,37,88,89]. In agreement with the changes to microbial taxa and with previous iron-metabolite studies [90], we found that iron increased cecal concentrations of acetate, butyrate, and propionate (Figure 4)—all three of these SCFAs have important roles in host-microbe interactions. Microbial-derived butyrate, produced by *Clostridia*, is essential for T_reg_ cell differentiation in the colon and protects against colitis [88]. Butyrate and propionate derived from soluble fiber improve metabolic function in a gut-brain communication loop [89]. Conversely, microbial acetate elevated in the context of a high-fat diet promotes metabolic syndrome by activating the parasympathetic nervous system to stimulate ghrelin and insulin secretion [38]. However, acetate produced by *Bifidobacteria* protected mice from enterotoxic *E. coli* [34]. Our results that oral iron increases SCFA production indicate several possible host-microbe crosstalk mechanisms by which iron supplementation affects development.

It is crucial to note that cecal metabolites following iron supplementation depended on iron form. Although both iron forms increased cecal acetate, only FS increased butyrate, propionate, and isovalerate (a branched SCFA), and only FC increased TMA, a metabolite that is associated with cardiovascular health. These differences reflect delineating alterations in microbial taxonomy between iron treatments. Compared to CON, the relative abundance of the butyrate-producer *Ruminococcus* was higher in the FS group, consistent with the elevation of butyrate in the FC group. Conversely, other producers of SCFAs (e.g., *Akkermansia*) were higher after FC treatment, which generated acetate, not butyrate or propionate. Moreover, levels of Firmicutes, known producers of TMA [91,92], were lower, but TMA was elevated in the FC group compared to CON. Iron serves as a cofactor for numerous metabolic reactions in both humans and microorganisms. Therefore, these metabolite differences may be reflective of both microbial and host metabolic function, as modulated via iron availability.

It is unclear how FC and FS exert differential effects, but it is possible that these iron forms are absorbed and metabolized by bacteria with different efficiencies. Consistent with our study, differences in microbiome composition were found previously between FS and FC iron groups; luminal iron levels were elevated but comparable between iron groups, challenging the idea that microbiome differences between forms arose from differences in iron availability [76]. In our study, differential effects of FS and FC on alpha-diversity were found in YA rats. A recent study in adult mice found that microbiome Shannon diversity effects differed according to iron form—FS or Sucrosomial^®^ iron—but observed that species richness increased similarly. A reduction in *Lactobacillaceae* was observed only in Sucrosomial^®^ iron-treated mice [75].

Exactly how iron supplementation alters the gut microbiota is also unclear. Previous experiments have shown that oral iron increases iron availability in the gastrointestinal lumen, and this may have direct or indirect effects on the gut microbiota. Certain bacteria, such as *Lactobacillus*, do not require iron, and this provides a competitive advantage in low-iron environments, while others, such as *E. coli*, gain an ecological niche in high-iron conditions [93,94]. The aforementioned study also reported that increasing dietary iron in adult mice increased luminal iron, shifted microbial communities and metabolism, and reduced the abundance of *Lactobacillus* [76]. Likewise, our data support the idea that *Lactobacillus*, which does not require iron for growth, is sensitive to exogenous iron in the pre-weanling gut.

Iron may affect the gut microbial community indirectly by affecting distal small intestine gene expression: Il22, a major regulator of mucosal immunity [57], was suppressed in iron-treated intestine (Figure 1C). To our knowledge, no studies have measured intestinal Il22 regulation in nursing rats treated with oral iron. It is possible that distal intestine iron loading may have altered the microbiota by causing a shift in mucosal immunity and antimicrobial activity [57,60]. Another possibility is that the change in Il22 expression was mediated by microbes, suggesting that iron affects host immunity by altering the gut microbiota. *Lactobacillus* bacteria upregulate Il22 in the intestine [35,36,95]; therefore, iron may cause lower intestinal Il22 expression by reducing the abundance of *Lactobacilli* in the distal intestine. In the small intestine, Il22 expression increases rapidly with weaning and is relatively low during the pre-weaning period compared to adult expression [96]. The functional impact of iron suppressing Il22 expression in the intestine during this developmental period remains unclear.

Our pre-weanling rat supplementation model has several strengths that increase relevance to human infant nutrition. Our dose is well within the physiological range and was specifically designed to represent the average iron intake from iron formula in a healthy, term infant [39]. Similar to human infants [28], iron absorption in rat pups is unregulated during early infancy [97], but it becomes regulated in late infancy. Further, the mechanisms regulating iron absorption during early life are similar in humans and in rat pups [98]. The rat litters were culled to control for growth and separated by treatment to avoid coprophagic iron/microbiota transfer. We used a moderate sample size of outbred rats—at least three litters for each treatment, allowing for assessment of litter and sex variables. There are also several strengths inherent to our microbiome assessment methods compared to previous studies in this area. We identified amplicon sequence variants as opposed to operational taxonomic units, allowing for more precise and reproducible identification of microbes [99]. Additionally, while several prior studies measured differential abundance based on hand-selected taxa, we characterized all differentially abundant taxa associated with iron treatment. A key limitation of our study is that we did not perform longitudinal measurements of the microbiome, which will be necessary to confirm microbiome changes in adulthood that are contributable to iron supplementation in early life.

## 5. Conclusions

Our results support prior work demonstrating that postnatal iron supplementation alters gut microbiota development. Our study demonstrates that microbiome changes due to iron supplementation in pre-weanling rats depend on the form of supplemental iron, and shows that iron supplementation in early life results in long-term alterations in the microbiome of adult rats. The functional consequences of these gut microbiome changes remain to be elucidated.

## Data Availability

The data presented in this study are not publicly available. The data and full reproducible code are available on request from the corresponding author.

## Supplementary Materials

The following supporting information can be downloaded at: https://www.mdpi.com/2072-6643/14/3/412/s1, Figure S1: Distal small intestine morphology following FS or FC supplementation at PD 15, Figure S2: Additional alpha-diversity and beta-diversity measures at PD 15, Figure S3: Additional cecal metabolite data, Figure S4: Additional alpha-diversity and beta-diversity measures in YA rats, Figure S5: Additional alpha-diversity & beta-diversity measures in YA rats, Figure S6: YA FC vs. FS differential abundance at phylum and genus levels, Table S1: Primer sequences for PCR, Table S2: *p*-values from diversity statistical analyses at PD 15, Table S3: Phyla differential abundance group comparisons at PD 15, Table S4: Genera differential abundance group comparisons at PD 15, Table S5: Iron status and weight of YA rats following daily postnatal iron supplementation with FS or FC, Table S6: *p*-values from diversity statistical analyses in YA rats, Table S7: Young adult rat phyla differential abundance supplementation group comparisons, Table S8: Young adult rat genera differential abundance supplementation group comparisons.
