# Correction: McMillen et al. Gut Microbiome Alterations following Postnatal Iron Supplementation Depend on Iron Form and Persist into Adulthood. *Nutrients* 2022, *14*, 412

**DOI:** 10.3390/nu14173585

**Published:** 2022-08-31

**Authors:** Shasta McMillen, Sydney Thomas, Emily Liang, Eric B. Nonnecke, Carolyn Slupsky, Bo Lönnerdal

**Affiliations:** 1 Department of Nutrition, University of California, Davis, CA 95616, USA; 2 Department of Food Science, University of California, Davis, CA 95616, USA

**Error in Figures and Captions**

In the original publication [1], there was a mistake in Figure 2D, Figure 3, Figure 5D, and Figure 6, and Supplementary Figures S3 and S6 and their captions, as published. The x-axes in these figures were mislabeled as Fold-Change instead of Log_2_[Fold-Change]. The corrected Figure 2D, Figure 3, Figure 5D, and Figure 6, and Supplementary Figures S3 and S6 appear below. 

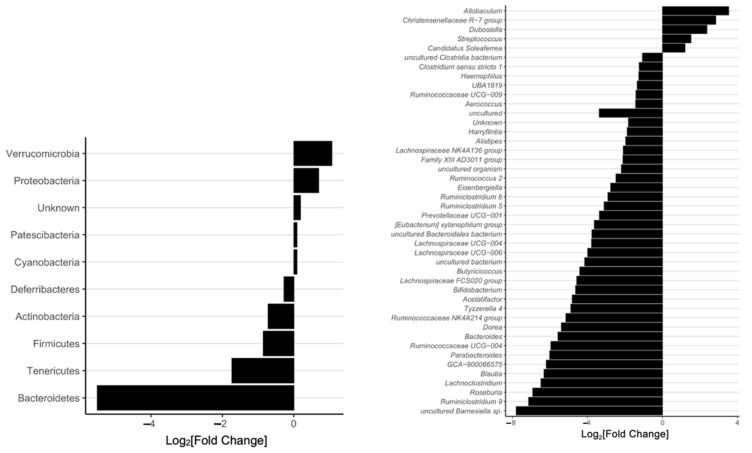



**Figure S3.** Differential abundance of cecal bacteria at the phylum (**left**) and genus level (**right**) in FC vs. FS treated pups at PD 15. All genera plotted are significantly different between iron groups. Differential abundance was determined using DESeq2 and FDR-adjusted *p*-values < 0.05 from pairwise group comparisons were considered significant. *p*-values are listed in Tables S3 and S4.

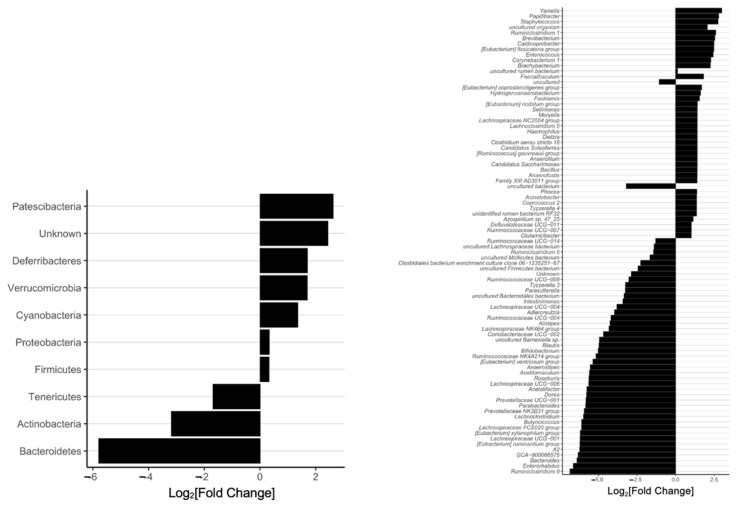



**Figure S6.** Differential abundance of cecal bacteria at the phylum (**left**) and genus level (**right**) in YA rats postnatally supplemented with FC vs. FS. All genera plotted are significantly different between iron groups. Differential abundance was determined using DESeq2 and FDR-adjusted *p*-values < 0.05 from pairwise group comparisons were considered significant. *p*-values are listed in Tables S7 and S8.

**Text Correction**

There was an error in the original publication. The fold-change values of differential taxa abundance were not converted from Log_2_[Fold Change]. Corrections have been made to the following sections:

Abstract:

“Iron provision resulted in 10,000-fold reduced abundance of *Lactobacilli* in pre-weanling and YA animals provided iron in early life (*p* < 0.0001).”

Methods, Section 2.8, Paragraph 4:

“Pairwise results were extracted and plotted as log_2_[Fold Change], and FDR-adjusted…”

Results, Section 3.2.3, Paragraph 1:

“In FC-treated pups we found a 100-fold lower abundance of Bacteroidetes…; Verrucomicrobia were over 6-fold elevated (*p* < 0.001).”

Results, Section 3.2.3, Paragraph 3:

“Iron-treated pups had a 10,000-fold lower abundance of *Lactobacilli* compared to CON pups (*p* < 0.0001); this effect was the largest we observed in all the 73 differentially abundant genera. *Bifidobacteria* were also less abundant in iron-treated pups (*p* < 0.05), as were *Turicibacter* (*p* < 0.0001), while *Ruminococcus 2* relative abundance was elevated (*p* < 0.0001). Bacteroides and Parabacteroides were 100-fold less abundant (*p* < 0.0001) in FC compared to CON, but these were uninfluenced by FS treatment.”

Results, Section 3.4.4, Paragraph 1:

“A lower abundance (~10-fold) of Proteobacteria was found in YA rats…”

Results, Section 3.4.4, Paragraph 2: 

“Consistent with results in pups, iron-treated rats had more than 10,000-fold lower abundance of *Lactobacillus* compared to CON rats (*p* < 0.0001)—the largest effect observed in all 95 differentially abundant genera in YA rats. *Turicibacter* abundance was 1000-fold lower in iron-treated rats (*p* < 0.0001). In iron-treated YA rats, but not in pups, abundance of *Escherichia-shigella* was around 10-fold lower than CON (*p* < 0.0001), and this effect was similar between FS and FC. Also consistent with pups, *Bifidobacterium* abundance in FC rats was 100-fold lower than in CON (*p* < 0.0001), although it was similar between FS and CON; *Bacteroides* abundance was 100-fold lower in FC rats compared to CON (*p* < 0.0001) and was comparable between FS and CON.”

The authors apologize for any inconvenience caused and state that the scientific conclusions are unaffected. The original publication has also been updated.

## Figures and Tables

**Figure 2:**
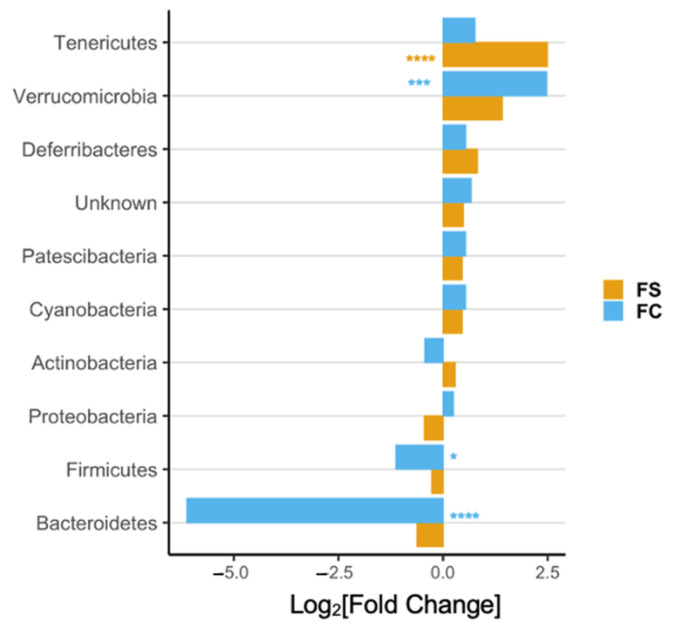
(**D**). Differential phyla abundance, represented as log_2_[Fold Change] from CON for each iron treatment group (*n* = 27–29/group, 3 litters/group); phyla are ordered by magnitude of change and bars are labeled with color according to iron treatment group.

**Figure 3:**
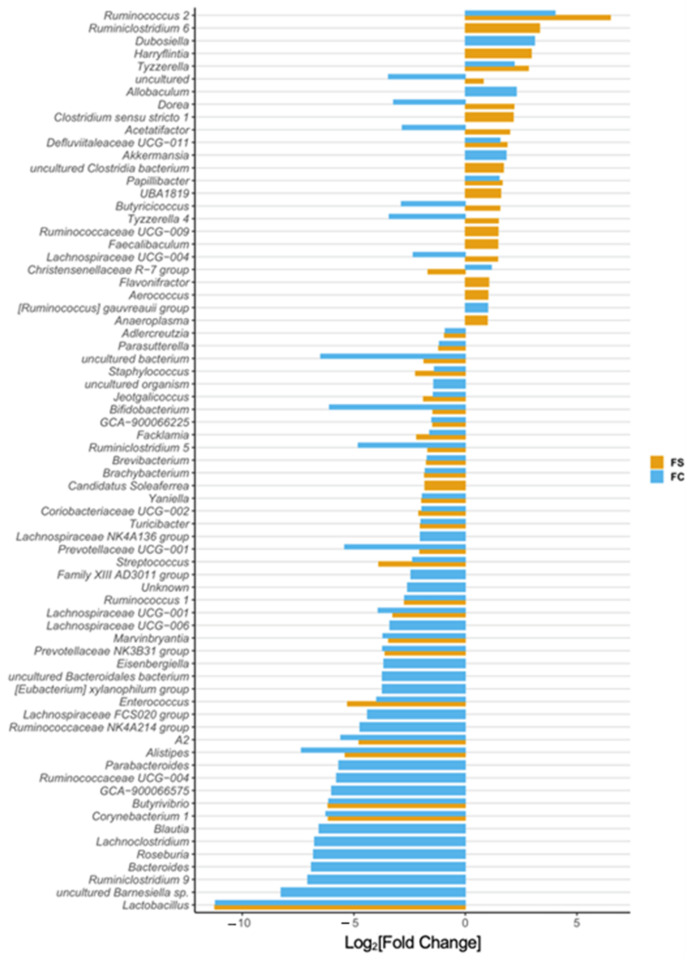
Differentially abundant genera due to iron form (*n* = 27–29/group, 3 litters/group). Differentially abundant genera are represented as log_2_[Fold Change] from CON for each iron group. Genera are ordered by magnitude of change. Differential abundance was assessed with DESeq2, and FDR-adjusted *p*-values < 0.05 were considered significant. All significant results are shown in the plot, and their adjusted *p*-values are listed in Table S4.

**Figure 5:**
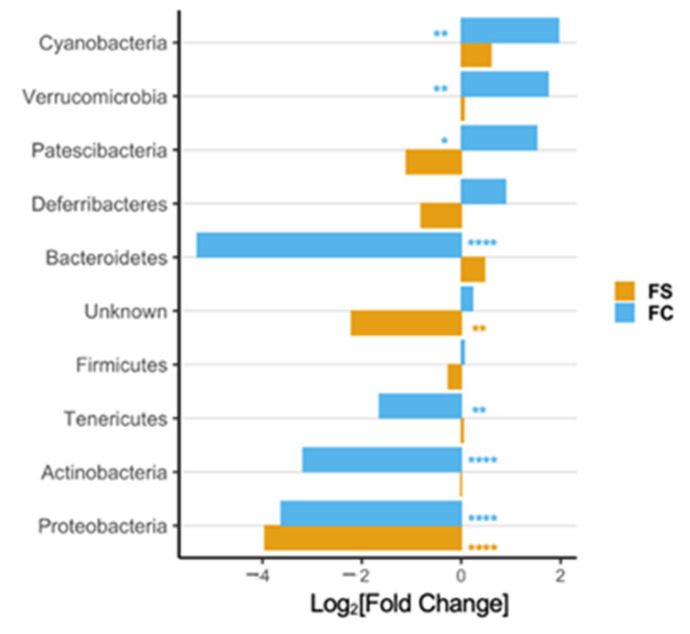
(**D**) Differential phyla abundance, represented as log_2_[Fold Change] from CON for each iron group (*n* = 19–23/group, 4 litters/group); phyla are ordered by magnitude of change and bars are labeled with color according to iron group.

**Figure 6:**
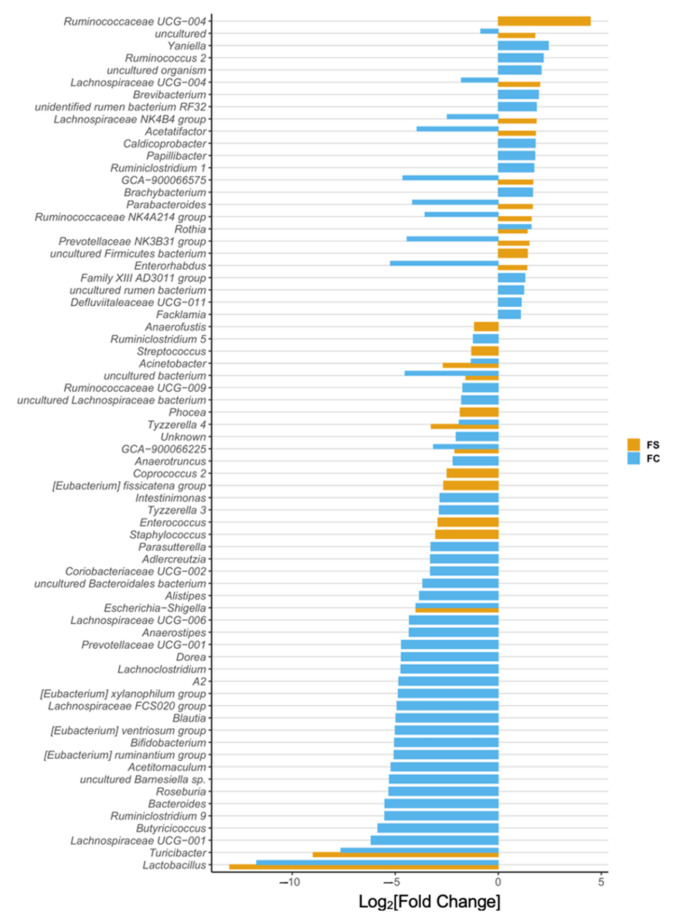
Differentially abundant genera in young adult (YA) rats due to iron form (*n* = 19–23/group, 4 litters/group). Differentially abundant genera are represented as log_2_[Fold Change] from CON for each iron group. Genera are ordered by magnitude of change. Differential abundance was assessed with DESeq2, and FDR-adjusted *p*-values < 0.05 were considered significant. All significant comparison results are shown in the plot, and their adjusted *p*-values are listed in Table S8.
