# Supplementary material for: Gut Microbiome Alterations following Postnatal Iron Supplementation Depend on Iron Form and Persist into Adulthood
Source: Nutrients. 2022 Jan 18;14(3):412. doi: 10.3390/nu14030412 (PMC8838803; doi:10.3390/nu14030412)
Supplement: Supplementary file 1 [file nutrients-14-00412-s001.zip › nutrients-1525383-supplementary.pdf]

Supplementary Information

Gut microbiome alterations following postnatal iron supplementation depend on iron form and persist into adulthood

Shasta McMillen, Sydney Thomas, Emily Liang, Eric B. Nonnecke, Carolyn Slupsky, and Bo Lönnerdal*

Supplementary Figure S1. Distal small intestine morphology following FS or FC supplementation at PD 15.
Supplementary Figure S2. Additional alpha-diversity & beta-diversity measures at PD 15.
Supplementary Figure S3. PD 15 FC vs. FS differential abundance at phylum and genus levels
Supplementary Figure S4. Cecal metabolites not affected by iron treatment or iron form.
Supplementary Figure S5. Additional alpha-diversity & beta-diversity measures in YA rats.
Supplementary Figure S6. YA FC vs. FS differential abundance at phylum and genus levels
Supplementary Table S1. Primer sequences
Supplementary Table S2. P-values from diversity statistical analyses at PD 15.
Supplementary Table S3. Phylum differential abundance group comparisons at PD15.
Supplementary Table S4. Genus differential abundance group comparisons at PD15.
Supplementary Table S5. Iron status and weight of young adult (YA) rats.
Supplementary Table S6. P-values from diversity statistical analyses in YA rats.
Supplementary Table S7. YA rat Phylum differential abundance group comparisons.
Supplementary Table S8. YA rat Genus differential abundance group comparisons.

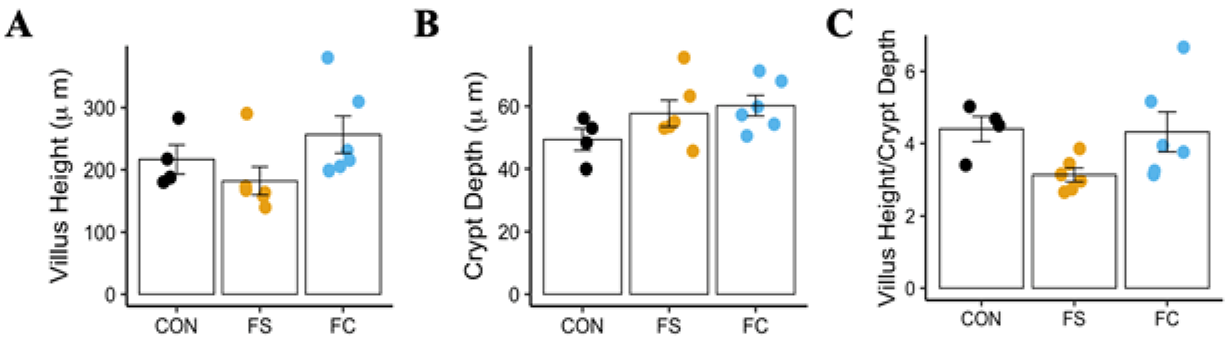

Supplementary Figure S1. Ileal morphology following FS or FC supplementation at PD 15. Ileal morphology was assessed in fixed, H&E-stained sections (n = 6/group, 3 litters/group). Mean A) villus height and B) crypt depth were calculated from 10 technical replicate measurements per sample/biological replicate. C) A ratio of villus height/crypt depth is also included.

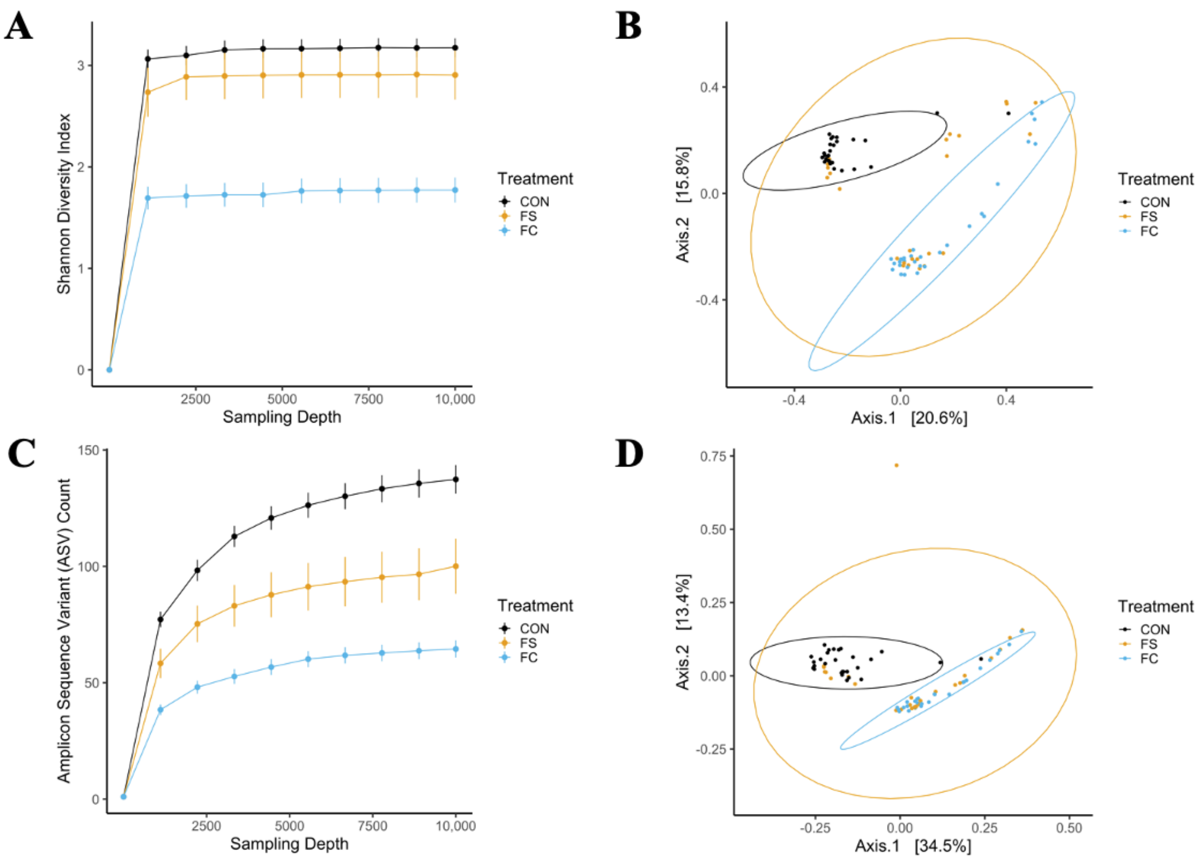

Supplementary Figure S2. Additional alpha-diversity & beta-diversity measures at PD 15. A) Alpha-diversity rarefaction plot depicting Shannon diversity by treatment group and sampling depth (n = 20-29/(group x sampling depth), n = 3 litters/group). Shannon index was decreased in both FS and FC groups compared to the CON at all sampling depths > 1. B) Principal coordinate analysis of Bray-Curtis distances, labeled by treatment group: all groups were dissimilar from each other by PERMANOVA (n = 27-29/group, 3 litters/group). C) Alpha-diversity rarefaction plot depicting Amplicon Sequence Variant (ASV) count (i.e., richness) by treatment group and sampling depth (n = 20-29/(group x sampling depth), n = 3 litters/group). ASV count decreased in both iron groups compared to the CON at all sampling depths > 1. D) Principal coordinate analysis of unweighted UniFrac distances, labeled by treatment group: all groups were dissimilar from each other by PERMANOVA (n = 27-29/group, 3 litters/group). Repeated Kruskal-Wallis tests with Dunn's multiple comparisons were used to test for differences in Shannon and ASV count among groups at each sampling depth. A PERMANOVA test was applied to detect microbiome compositional dissimilarity among treatment groups, using a nested model, with litter nested within treatment factor to account for litter effects.

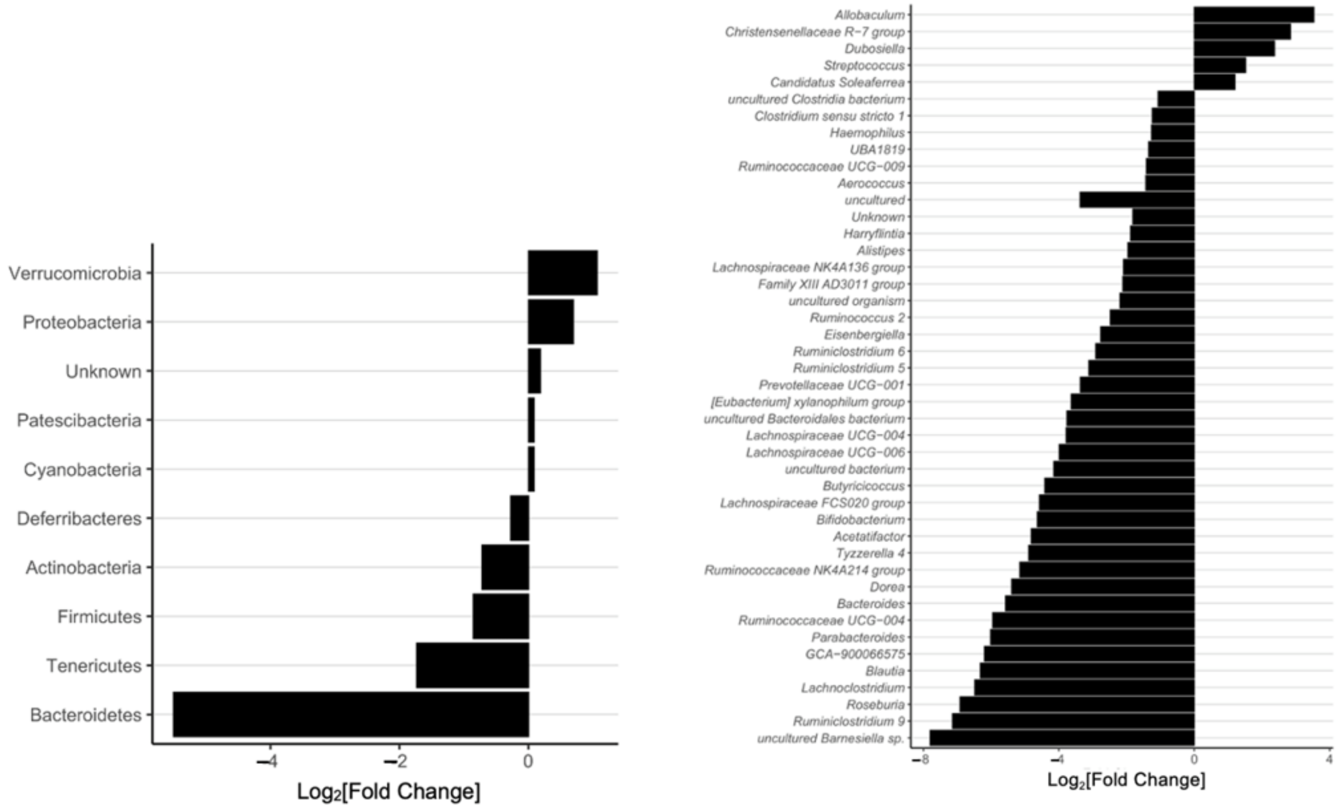

Supplementary Figure S3. Differential abundance of cecal bacteria at the phylum (left) and genus level (right) in FC vs. FS treated pups at PD 15. All genera plotted are significantly different between iron groups. Differential abundance was determined using DESeq2 and FDR-adjusted p-values < 0.05 from pairwise group comparisons were considered significant. P-values are listed in Tables S3-4.

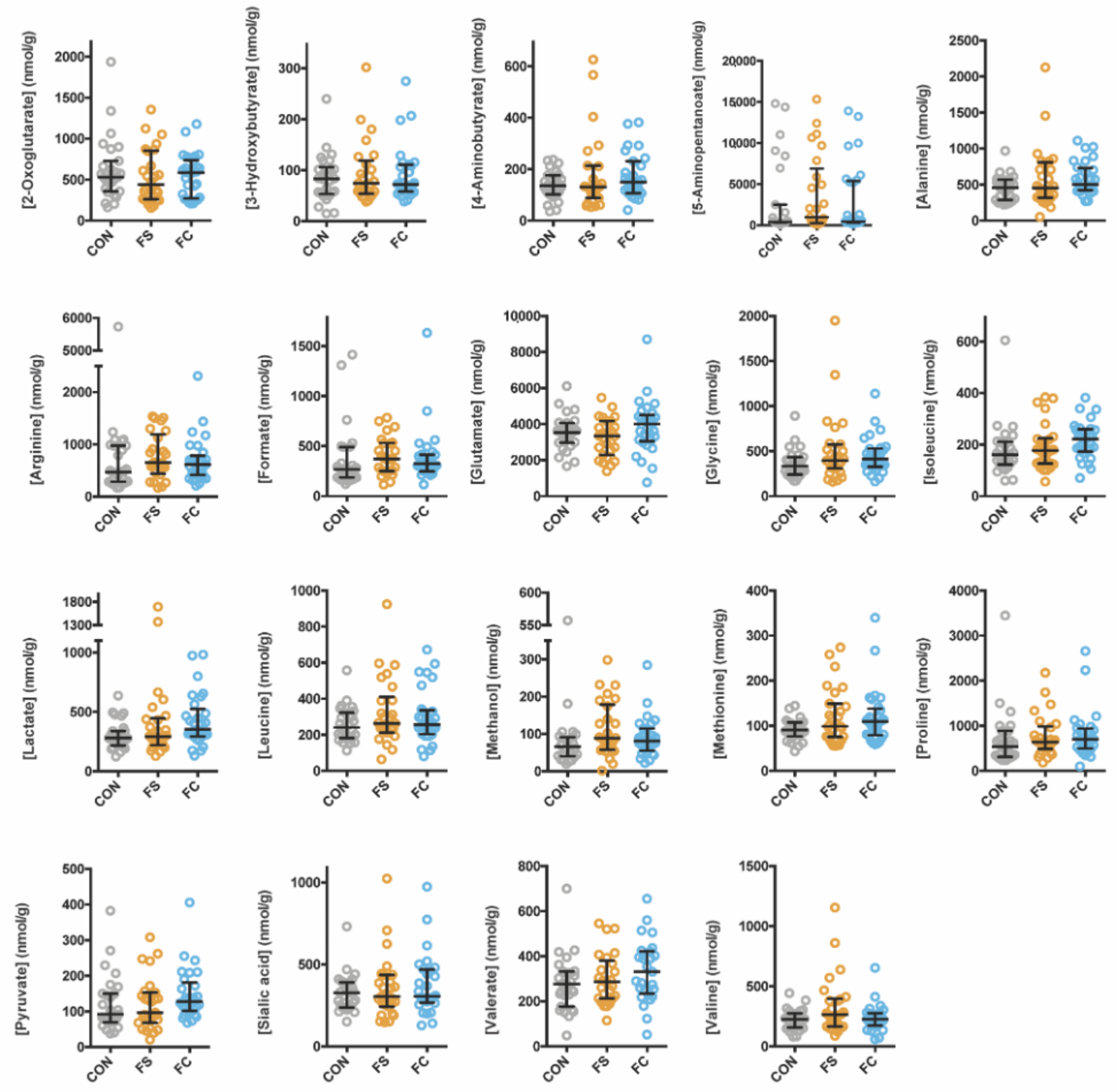

Supplementary Figure S4. Cecal metabolites detected in pups that were not affected by iron treatment or iron form. Metabolites were analyzed by Kruskal-Wallis followed by Dunn's multiple comparison testing. All metabolites shown are significant by Kruskal-Wallis. Median and interquartile range of concentrations are indicated (n = 27-29/group, 3 litters/group).

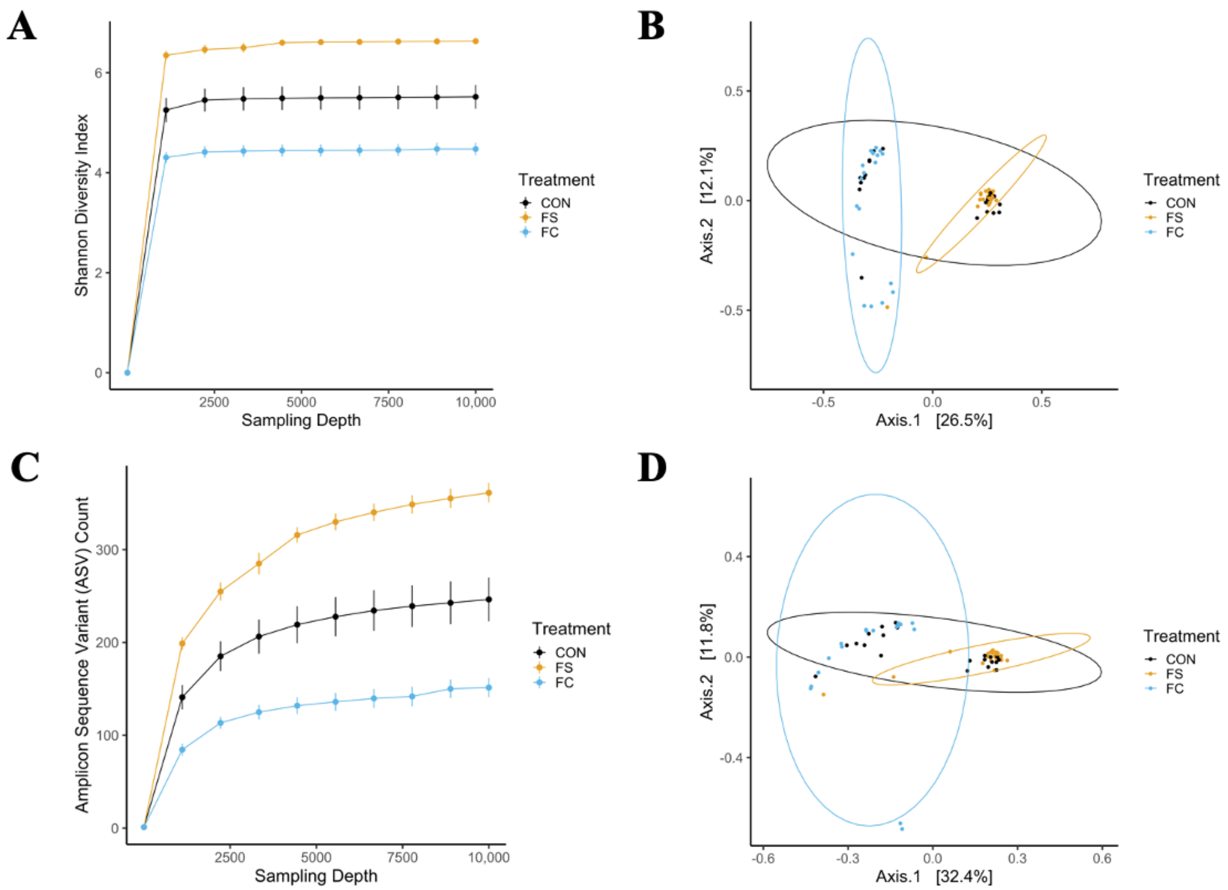

Supplementary Figure S5. Additional alpha-diversity & beta-diversity measures in YA rats. A) Alpha-diversity rarefaction plot depicting Shannon diversity by treatment group and sampling depth (n = 12-23/(group x sampling depth), 4 litters/group). Shannon index was increased in the FS group and decreased in the FC group compared to the CON at all sampling depths > 1. B) Principal coordinate analysis of Bray-Curtis distances, labeled by treatment group: all groups were dissimilar from each other by PERMANOVA (n = 19-23/group, 4 litters/group). C) Alpha-diversity rarefaction plot depicting Amplicon Sequence Variant (ASV) count (i.e., richness) by treatment group and sampling depth (n = 12-23/(group x sampling depth), 4 litters/group). ASV count was increased in the FS group and decreased in the FC group compared to the CON at all sampling depths > 1. D) Principal coordinate analysis of unweighted UniFrac distances, labeled by treatment group: all groups were dissimilar from each other by PERMANOVA (n = 19-23/group, 4 litters/group). Repeated Kruskal-Wallis tests with Dunn's multiple comparisons were used to test for differences in Shannon and ASV count among groups at each sampling depth. A PERMANOVA test was applied to detect microbiome compositional dissimilarity among treatment groups, using a nested model, with litter nested within treatment factor to account for litter effects.

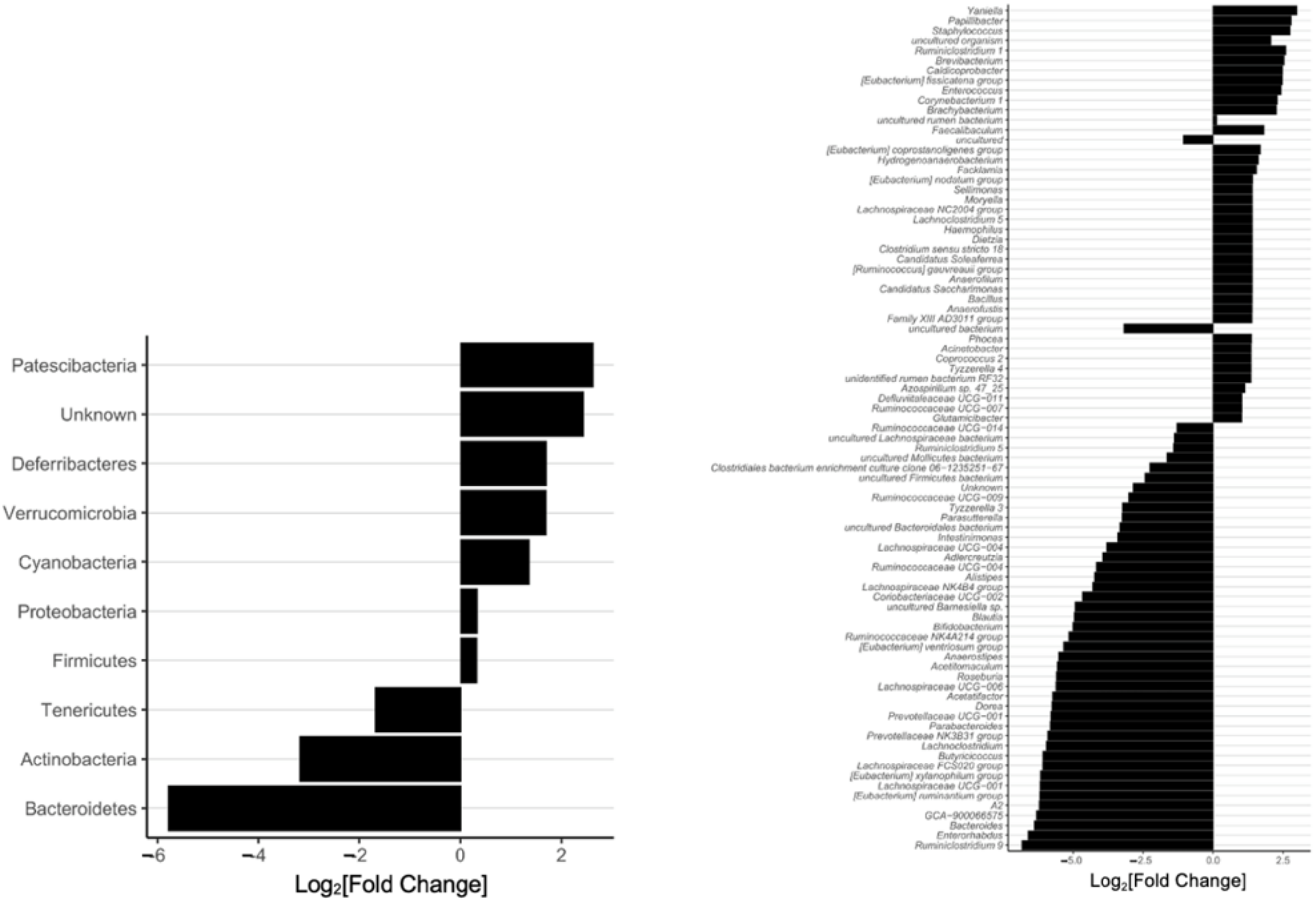

Supplementary Figure S6. Differential abundance of cecal bacteria at the phylum (left) and genus level (right) in YA rats postnatally supplemented with FC vs. FS. All genera plotted are significantly different between iron groups. Differential abundance was determined using DESeq2 and FDR-adjusted p-values < 0.05 from pairwise group comparisons were considered significant. P-values are listed in Tables S7-8.

Supplementary Table S1. Primer sequences for real-time PCR and 16s rRNA library construction.

Gene	Primer Sequence
Actb Forward[1]	5'-GAAATCGTGCGTGACATTAAAGAG -3
Actb Reverse[1]	5'-GCGGCAGTGGCCATCTC-3'
Il-22 Forward	5'- ATGCTCTGCCCATCAACTC -3'
Il-22 Reverse	5'- GCAGAACATCTTCAAGGGTG - 3'
Lcn2 Forward	5'-CAGAAAGAAAGACAAAGCCG-3'
Lcn2 Reverse	5'-TGGCAAACTGGTCGTAGTC-3'
Lyz Forward	5′-AGGAATGGGATGTCTGGCTAC-3′
Lyz Reverse	5′-GGTATCCCACAGGCGTTCTT-3′
Reg3g Forward	5'-ATGCCAAGGAAGATGTGCCCAC-3'
Reg3g Reverse	5'-AATCAAGGAGGACACAAAGG-3'
Gpx4 Forward[2]	5`-CAGCAAGATCTGTGTAAATGGGG- 3`
Gpx4 Reverse[2]	5`-CTTGGTGAAGTTCCATTTGATGG- 3`
Nox4 Forward[3]	5′-GTGAACGCCCTGAACTTCTC-3′
Nox4 Reverse[3]	5′-TTCTGGGATCCTCATTCTGG-3′
p22^{phox} Forward[3]	5′-TGTTGCAGGAGTGCTCATCTGTCT-3′
p22^{phox} Reverse[3]	5′-AGGACAGCCCGGACGTAGTAATTT-3′
16s rRNA V4 Forward[#]	5'- AATGATACGGCGACCACCGAGATCTACACTCTTTCCCTACACGACGCTCTTCCGATCTNNNNNNNN*GTGTGCCAGCMGCCGCGGTAA-3'
16s rRNA V4 Reverse[#]	5'- CAAGCAGAAGACGGCATACGAGATCGGTCTCGGCATTCCTGCTGAACCGCTCTTCCGATCTCCGGACTACHVGGGTWTCTAAT-3'

[#]Full primer sequence with Illumina adaptor sequencing primer, barcode (forward only), linker, and PCR primer; *8-Nucleotide Barcode [1] Li, Y.; Yu, P.; Chang, S.-Y.; Wu, Q.; Yu, P.; Xie, C.; Wu, W.; Zhao, B.; Gao, G.; Chang, Y.-Z. Hypobaric Hypoxia Regulates Brain Iron Homeostasis in Rats. *J. Cell. Biochem.* **2017**, 118, 1596–1605. https://doi.org/10.1002/jcb.25822. [2] Sukhanova, I.A.; Sebentsova, E.A.; Khukhareva, D.D.; Manchenko, D.M.; Glazova, N.Y.; Vishnyakova, P.A.; Inozemtzeva, L.S.; Dolotov, O.V.; Vyokikh, M.Y.; Levitskaya, N.G. Gender-Dependent Changes in Physical Development, BDNF Content and GSH Redox System in a Model of Acute Neonatal Hypoxia in Rats, *Behavioural Brain Research*, **2018**, 350, 87-98. https://doi.org/10.1016/j.bbr.2018.05.008. [3] Cavdar, Z.; Oktan, M.A.; Ural, C.; Calisir, M.; Kocak, A.; Heybeli, C.; Yildiz, S.; Arici A.; Ellidokuz, H.; Celik, A.; Yilmaz, O.; Sarioglu, S.; Cavdar, C. Renoprotective Effects of Alpha Lipoic Acid on Iron Overload-Induced Kidney Injury in Rats by Suppressing NADPH Oxidase 4 and p38 MAPK Signaling. *Biol Trace Elem Res* **193,** 483–493 (2020). https://doi.org/10.1007/s12011-019-01733-3.

Supplementary Table S2. P-values from diversity statistical analyses at PD 15.

Alpha-Diversity Metric (Sampling Depth)	P-Value			
**Faith's PD[1]**	**Treatment[2]**	**FS vs. CON[3]**	**FC vs. CON[3]**	FC vs. FS[3]
(1)	0.1108	0.0347	0.0346	0.4871
(1112)	<0.0001	0.0013	<0.0001	0.0075
(2223)	<0.0001	0.0028	<0.0001	0.0058
(3334)	<0.0001	0.0011	<0.0001	0.0059
(4445)	<0.0001	<0.0001	<0.0001	0.0078
(5556)	<0.0001	0.0006	<0.0001	0.0126
(6667)	<0.0001	0.0007	<0.0001	0.0123
(7778)	<0.0001	0.0006	<0.0001	0.0144
(8889)	<0.0001	0.0005	<0.0001	0.0169
(10,000)	<0.0001	0.0011	<0.0001	0.0104
Shannon[4]	Treatment[2]	FS vs. CON[3]	FC vs. CON[3]	FC vs. FS[3]
(1)	NA			
(1112)	<0.0001	0.0507	<0.0001	0.0001
(2223)	<0.0001	0.0966	<0.0001	<0.0001
(3334)	<0.0001	0.0741	<0.0001	0.0001
(4445)	<0.0001	0.0782	<0.0001	0.0001
(5556)	<0.0001	0.0762	<0.0001	0.0001
(6667)	<0.0001	0.0741	<0.0001	0.0001
(7778)	<0.0001	0.0670	<0.0001	0.0001
(8889)	<0.0001	0.0672	<0.0001	0.0001
(10,000)	<0.0001	0.0702	<0.0001	0.0001
ASVs[5]	Treatment[2]	FS vs. CON[3]	FC vs. CON[3]	FC vs. FS[3]
(1)	NA			
(1112)	<0.0001	0.0036	<0.0001	0.0041
(2223)	<0.0001	0.0049	<0.0001	0.0038
(3334)	<0.0001	0.0013	<0.0001	0.0056
(4445)	<0.0001	0.0012	<0.0001	0.0084
(5556)	<0.0001	0.0012	<0.0001	0.0118
(6667)	<0.0001	0.0012	<0.0001	0.0133
(7778)	<0.0001	0.0013	<0.0001	0.0128
(8889)	<0.0001	0.0010	<0.0001	0.0150
(10,000)	<0.0001	0.0021	<0.0001	0.0109

	**P-Value[6]**				
**Beta-Diversity Metric**	Treatment	Sex	Treatment: Litter	Treatment: Sex	Treatment: Sex:Litter
Weighted UniFrac	0.0806	0.9027	0.4188	0.4792	0.1542
Unweighted UniFrac	<0.0001	0.1910	<0.0001	0.0094	<0.0001
Bray-Curtis	<0.0001	0.1643	<0.0001	0.0631	0.0762

[1] Faith's Phylogenetic Diversity. [2] Kruskal-Wallis test for treatment effect. [3] Dunn's pairwise group comparisons. [4] Shannon's Diversity. [5] Amplicon Sequence Variant count (richness). [6] Permutational analysis of variance (PERMANOVA)

Supplementary Table S3. Phylum differential abundance group comparisons at PD15.

Phylum	Comparison	baseMean	log2FoldChange	padj
Actinobacteria	FS_vs_CON	703.827659	0.28597037	0.58918018
Bacteroidetes	FS_vs_CON	2635.15429	-0.6223581	0.34548905
Cyanobacteria	FS_vs_CON	1.71118619	0.45257065	0.33812189
Deferribacteres	FS_vs_CON	1.79451952	0.81560873	0.13738362
Firmicutes	FS_vs_CON	8568.86702	-0.2698228	0.5888096
Patescibacteria	FS_vs_CON	1.71118619	0.45257065	0.33812189
Proteobacteria	FS_vs_CON	20396.4905	-0.4459152	0.33812189
Tenericutes	FS_vs_CON	2.81411863	2.49124336	2.44E-06
Verrucomicrobia	FS_vs_CON	1195.09706	1.41225889	0.11346409
Unknown	FS_vs_CON	1.73555216	0.48361737	0.33812189
Actinobacteria	FC_vs_CON	703.827659	-0.4331528	0.45001362
Bacteroidetes	FC_vs_CON	2635.15429	-6.1216826	1.58E-26
Cyanobacteria	FC_vs_CON	1.71118619	0.53769121	0.22095926
Deferribacteres	FC_vs_CON	1.79451952	0.54169409	0.23724916
Firmicutes	FC_vs_CON	8568.86702	-1.123884	0.02578339
Patescibacteria	FC_vs_CON	1.71118619	0.53769121	0.22095926
Proteobacteria	FC_vs_CON	20396.4905	0.24616001	0.5060345
Tenericutes	FC_vs_CON	2.81411863	0.76021629	0.22095926
Verrucomicrobia	FC_vs_CON	1195.09706	2.47551078	0.00023836
Unknown	FC_vs_CON	1.73555216	0.66777386	0.22095926
Actinobacteria	FC_vs_FS	703.827659	-0.7191231	0.28484582
Bacteroidetes	FC_vs_FS	2635.15429	-5.4993245	3.85E-21
Cyanobacteria	FC_vs_FS	1.71118619	0.08512055	0.81817107
Deferribacteres	FC_vs_FS	1.79451952	-0.2739146	0.69203079
Firmicutes	FC_vs_FS	8568.86702	-0.8540612	0.14984137
Patescibacteria	FC_vs_FS	1.71118619	0.08512055	0.81817107
Proteobacteria	FC_vs_FS	20396.4905	0.69207522	0.15996684
Tenericutes	FC_vs_FS	2.81411863	-1.7310271	0.00105402
Verrucomicrobia	FC_vs_FS	1195.09706	1.06325189	0.16710452
Unknown	FC_vs_FS	1.73555216	0.18415649	0.79143125

Supplementary Table S4. Genus differential abundance group comparisons at PD15.

Genus	Comparison	baseMean	log2FoldChange	padj
Lactobacillus	FC_vs_CON	943.343916	-11.2054027	5.91E-53
Lactobacillus	FS_vs_CON	943.343916	-11.23517868	4.23E-51
Corynebacterium 1	FC_vs_CON	31.0486927	-6.257605456	4.13E-36
Corynebacterium 1	FS_vs_CON	31.0486927	-6.147020256	3.04E-34
Alistipes	FC_vs_CON	87.7495671	-7.351784331	3.77E-29
Roseburia	FC_vs_CON	843.041103	-6.810033651	3.77E-29
Ruminiclostridium 9	FC_vs_CON	137.932944	-7.068756355	4.41E-29
Roseburia	FC_vs_FS	843.041103	-6.918374768	4.81E-29
Ruminiclostridium 9	FC_vs_FS	137.932944	-7.145837222	7.57E-29
Lachnoclostridium	FC_vs_CON	129.069791	-6.763864109	5.03E-28
uncultured Barnesiella sp.	FC_vs_CON	209.180772	-8.26164139	8.59E-28
Bacteroides	FC_vs_CON	691.280665	-6.90262688	2.49E-27
uncultured bacterium	FC_vs_CON	194.772508	-6.487304064	2.92E-26
Butyrivibrio	FC_vs_CON	28.5753277	-6.129347333	4.94E-26
Lachnoclostridium	FC_vs_FS	129.069791	-6.489045554	4.26E-25
Prevotellaceae UCG−001	FC_vs_CON	21.1685806	-5.41264579	4.84E-25
GCA−900066575	FC_vs_FS	53.9507492	-6.202951435	7.53E-25
Butyrivibrio	FS_vs_CON	28.5753277	-6.160081634	1.15E-24
uncultured Barnesiella sp.	FC_vs_FS	209.180772	-7.806397155	2.58E-24
GCA-900066575	FC_vs_CON	53.9507492	-5.997231736	4.64E-24
A2	FC_vs_CON	20.1290152	-5.585794341	1.14E-22
Parabacteroides	FC_vs_FS	2137.90368	-6.018782706	3.01E-21
Blautia	FC_vs_CON	1069.40878	-6.560698938	3.17E-21
Enterococcus	FS_vs_CON	16.4559886	-5.283282694	2.00E-20
Parabacteroides	FC_vs_CON	2137.90368	-5.676176076	1.07E-19
uncultured	FC_vs_CON	394.333165	-5.389424988	1.07E-19
Blautia	FC_vs_FS	1069.40878	-6.32235754	4.15E-19
Bifidobacterium	FC_vs_CON	37.1723205	-6.093653026	1.18E-18
Ruminococcaceae UCG−004	FC_vs_FS	74.2474539	-5.956391187	1.52E-18
Dorea	FC_vs_FS	20.2595329	-5.400013303	2.36E-18
Ruminococcaceae UCG−004	FC_vs_CON	74.2474539	-5.779379862	4.00E-18
Ruminiclostridium 5	FC_vs_CON	88.7515121	-4.804650556	4.94E-18
Bacteroides	FC_vs_FS	691.280665	-5.575954943	9.17E-18
Ruminococcus 2	FS_vs_CON	321.495762	6.504726938	9.99E-18
A2	FS_vs_CON	20.1290152	-4.769059001	1.82E-17
Alistipes	FS_vs_CON	87.7495671	-5.391805283	4.24E-17
uncultured	FC_vs_FS	394.333165	-5.047944312	6.71E-17
Ruminococcaceae NK4A214 group	FC_vs_FS	27.6491415	-5.153268129	5.71E-16
Lachnospiraceae FCS020 group	FC_vs_FS	21.8107195	-4.577687561	6.76E-16
Prevotellaceae NK3B31 group	FC_vs_CON	5.99282075	-3.713898082	7.36E-16
Lachnospiraceae FCS020 group	FC_vs_CON	21.8107195	-4.388253466	4.44E-15
Enterococcus	FC_vs_CON	16.4559886	-3.976861459	6.37E-15
Lachnospiraceae UCG−001	FC_vs_CON	6.94437535	-3.909293473	2.04E-14
Prevotellaceae NK3B31 group	FS_vs_CON	5.99282075	-3.602050531	2.20E-14
Acetatifactor	FC_vs_FS	14.0277087	-4.818296549	2.77E-14
Butyricicoccus	FC_vs_FS	11.4562801	-4.418002071	5.03E-14
Ruminococcaceae NK4A214 group	FC_vs_CON	27.6491415	-4.726550258	5.15E-14
Tyzzerella 4	FC_vs_FS	16.0396134	-4.892159519	1.02E-12
Lachnospiraceae UCG−006	FC_vs_FS	10.5895424	-3.990325886	1.78E-12
[Eubacterium] xylanophilum group	FC_vs_CON	10.361042	-3.72527298	4.28E-12
[Eubacterium] xylanophilum group	FC_vs_FS	10.361042	-3.638566341	3.06E-11
uncultured bacterium	FC_vs_FS	194.772508	-4.152684958	3.93E-11
Lachnospiraceae UCG−004	FC_vs_FS	7.67180536	-3.789248869	4.70E-11

Bifidobacterium	FC_vs_FS	37.1723205	-4.636273134	5.75E-11
Lachnospiraceae UCG−001	FS_vs_CON	6.94437535	-3.253299492	1.57E-10
Ruminiclostridium 6	FS_vs_CON	4.11142561	3.329217364	1.98E-10
Eisenbergiella	FC_vs_CON	7.9919944	-3.657999786	3.22E-10
Prevotellaceae UCG−001	FC_vs_FS	21.1685806	-3.369521952	5.56E-10
Marvinbryantia	FC_vs_CON	20.4290374	-3.698506609	5.58E-10
uncultured	FC_vs_CON	228.601474	-3.684153644	8.09E-10
Streptococcus	FS_vs_CON	91.7217949	-3.881505602	1.14E-09
Lachnospiraceae UCG−006	FC_vs_CON	10.5895424	-3.38531779	1.82E-09
Dubosiella	FC_vs_CON	22.0619511	3.102949782	2.06E-09
Ruminiclostridium 6	FC_vs_FS	4.11142561	-2.912986946	6.11E-09
uncultured Bacteroidales bacterium	FC_vs_CON	10.861042	-3.723859161	8.01E-09
uncultured Bacteroidales bacterium	FC_vs_FS	10.861042	-3.765574934	9.37E-09
uncultured	FC_vs_FS	228.601474	-3.498998978	9.80E-09
Harryflintia	FS_vs_CON	7.34161842	2.959963163	1.06E-08
Marvinbryantia	FS_vs_CON	20.4290374	-3.439243111	3.24E-08
Ruminiclostridium 5	FC_vs_FS	88.7515121	-3.119114705	5.66E-08
Ruminococcus 2	FC_vs_CON	321.495762	4.021804891	7.05E-08
Dorea	FC_vs_CON	20.2595329	-3.218620782	2.62E-07
Christensenellaceae R−7 group	FC_vs_FS	18.8476462	2.841349044	4.49E-07
Tyzzerella 4	FC_vs_CON	16.0396134	-3.413027173	7.57E-07
Allobaculum	FC_vs_FS	54.1839724	3.531318175	1.27E-06
Butyricicoccus	FC_vs_CON	11.4562801	-2.869376308	1.41E-06
Unknown	FC_vs_CON	647.718827	-2.593479496	2.52E-06
Turicibacter	FC_vs_CON	2.3501702	-1.991442032	2.99E-06
Coriobacteriaceae UCG−002	FC_vs_CON	2.87419557	-1.952481966	4.57E-06
Eisenbergiella	FC_vs_FS	7.9919944	-2.764685863	5.08E-06
Coriobacteriaceae UCG−002	FS_vs_CON	2.87419557	-2.089148552	5.62E-06
Dubosiella	FC_vs_FS	22.0619511	2.37203578	8.21E-06
Turicibacter	FS_vs_CON	2.3501702	-2.023357946	9.02E-06
Acetatifactor	FC_vs_CON	14.0277087	-2.82985385	1.13E-05
Yaniella	FC_vs_CON	2.28961344	-1.931604844	1.29E-05
uncultured	FS_vs_CON	136.226542	2.689102713	2.86E-05
Clostridium sensu stricto 1	FS_vs_CON	18.4608341	2.151358294	3.20E-05
Yaniella	FS_vs_CON	2.28961344	-1.963424876	3.35E-05
Brachybacterium	FC_vs_CON	2.15866106	-1.800938653	3.41E-05
Prevotellaceae UCG−001	FS_vs_CON	21.1685806	-2.043123838	3.75E-05
Facklamia	FS_vs_CON	5.7376221	-2.182870448	3.75E-05
Brevibacterium	FC_vs_CON	2.07664137	-1.713778527	4.29E-05
Ruminococcus 1	FC_vs_CON	152.19556	-2.72874267	4.34E-05
Tyzzerella	FS_vs_CON	39.6871385	2.827082801	5.00E-05
Family XIII AD3011 group	FC_vs_CON	16.92786	-2.443606159	6.16E-05
Staphylococcus	FS_vs_CON	46.7319341	-2.231779102	6.81E-05
Lachnospiraceae UCG−004	FC_vs_CON	7.67180536	-2.3397509	6.81E-05
Brachybacterium	FS_vs_CON	2.15866106	-1.832838099	6.86E-05
Brevibacterium	FS_vs_CON	2.07664137	-1.745791046	8.33E-05
Ruminococcus 1	FS_vs_CON	152.19556	-2.735077752	0.00010062
Streptococcus	FC_vs_CON	91.7217949	-2.368257518	0.00013857
Harryflintia	FC_vs_FS	7.34161842	-1.883044116	0.00018459
Lachnospiraceae NK4A136 group	FC_vs_FS	621.161071	-2.092981218	0.00018814
Lachnospiraceae NK4A136 group	FC_vs_CON	621.161071	-2.022065327	0.00019567
Dorea	FS_vs_CON	20.2595329	2.181392521	0.00028742
uncultured bacterium	FS_vs_CON	194.772508	-2.334619105	0.00037237
Candidatus Soleaferrea	FS_vs_CON	2.6256069	-1.812704475	0.00037237
uncultured Clostridia bacterium	FS_vs_CON	3.66684088	1.709144878	0.00079489

Family XIII AD3011 group	FC_vs_FS	16.92786	-2.11119267	0.00105365
Tyzzerella	FC_vs_CON	39.6871385	2.19266492	0.00111933
uncultured organism	FC_vs_FS	10.6022059	-2.199746717	0.00117072
Papillibacter	FS_vs_CON	4.69457407	1.655723721	0.00125534
Facklamia	FC_vs_CON	5.7376221	-1.602317825	0.0012586
Jeotgalicoccus	FS_vs_CON	76.1024512	-1.875900782	0.0013859
Acetatifactor	FS_vs_CON	14.0277087	1.988442699	0.00147479
Ruminococcus 2	FC_vs_FS	321.495762	-2.482922047	0.00175737
Allobaculum	FC_vs_CON	54.1839724	2.28574523	0.00176994
Unknown	FC_vs_FS	647.718827	-1.818557436	0.001928
Papillibacter	FC_vs_CON	4.69457407	1.515567825	0.00197424
Defluviitaleaceae UCG−011	FS_vs_CON	86.9538377	1.868385301	0.00210218
GCA−900066225	FC_vs_CON	7.71173761	-1.508080678	0.00271297
Aerococcus	FC_vs_FS	7.04821059	-1.432843032	0.0038711
Parasutterella	FC_vs_CON	1.67056582	-1.164849302	0.00528682
UBA1819	FS_vs_CON	8.90396681	1.588243399	0.0057122
Ruminiclostridium 5	FS_vs_CON	88.7515121	-1.685535851	0.00615359
Christensenellaceae R−7 group	FS_vs_CON	18.8476462	-1.673736317	0.00623561
GCA-900066225	FS_vs_CON	7.71173761	-1.465727826	0.00623561
Parasutterella	FS_vs_CON	1.67056582	-1.197023886	0.00708011
Butyricicoccus	FS_vs_CON	11.4562801	1.548625762	0.00742389
Defluviitaleaceae UCG−011	FC_vs_CON	86.9538377	1.553513294	0.00788842
Alistipes	FC_vs_FS	87.7495671	-1.959979049	0.00847069
Akkermansia	FC_vs_CON	1419.64758	1.829097439	0.01062134
Jeotgalicoccus	FC_vs_CON	76.1024512	-1.438837176	0.01091676
uncultured	FC_vs_CON	150.057441	-1.260348868	0.01091676
Staphylococcus	FC_vs_CON	46.7319341	-1.388924621	0.01098959
Lachnospiraceae UCG−004	FS_vs_CON	7.67180536	1.449497969	0.01122382
uncultured	FC_vs_FS	136.226542	-1.583467282	0.01796532
Clostridium sensu stricto 1	FC_vs_FS	18.4608341	-1.24537557	0.01976096
UBA1819	FC_vs_FS	8.90396681	-1.350481228	0.01976096
Ruminococcaceae UCG−009	FS_vs_CON	8.99034759	1.466312698	0.0198437
[Ruminococcus] gauvreauii group	FC_vs_CON	1.42236223	1.003701849	0.02096697
uncultured	FS_vs_CON	150.057441	-1.212349475	0.02299362
Flavonifractor	FS_vs_CON	1.43247059	1.047356077	0.02299362
Ruminococcaceae UCG−009	FC_vs_FS	8.99034759	-1.416327663	0.02428004
Streptococcus	FC_vs_FS	91.7217949	1.513248084	0.02970451
Candidatus Soleaferrea	FC_vs_FS	2.6256069	1.204416941	0.02970451
uncultured bacterium	FS_vs_CON	10.2376826	-1.382450735	0.0319048
Haemophilus	FC_vs_FS	5.0336956	-1.267429579	0.03330842
uncultured	FS_vs_CON	1.48303661	0.939428653	0.03348201
Adlercreutzia	FC_vs_CON	1.52770868	-0.905571756	0.03473611
Anaeroplasma	FS_vs_CON	1.56786663	0.985016645	0.03713742
uncultured organism	FC_vs_CON	10.6022059	-1.420277571	0.03801911
Faecalibaculum	FS_vs_CON	43.5243414	1.457861125	0.03859935
Tyzzerella 4	FS_vs_CON	16.0396134	1.479132346	0.0387226
Adlercreutzia	FS_vs_CON	1.52770868	-0.937801356	0.03926375
uncultured Clostridia bacterium	FC_vs_FS	3.66684088	-1.071782397	0.03968369
Christensenellaceae R−7 group	FC_vs_CON	18.8476462	1.167612727	0.04421831
Bifidobacterium	FS_vs_CON	37.1723205	-1.457379892	0.0481069
Aerococcus	FS_vs_CON	7.04821059	1.013597058	0.0481069

Supplementary Table S5. Iron status and weight of YA rats following daily postnatal iron supplementation with FS or FC.

	**CON**	**FS**	**FC**	**P-Value**
**Males**				
Hb (mg/L)	17.7 ± 1.4	17.4 ± 2.7	17.9 ± 0.8	0.8635
Fe (ppm)				
Liver	75.5 ± 14.7	76.9 ± 14.9	92.0 ± 14.7	0.1005
Spleen	166.7 ± 32.8	186.5 ± 50.8	171.6 ± 23.6	0.5303
Weight (g)				
Body	297.8 ± 29.46	303.3 ± 15.8	310.9 ± 15.0	0.3342
Liver	11.70 ± 0.95	12.47 ± 0.62	11.59 ± 1.4	0.1139
Brain	1.87 ± 0.11	1.89 ± 0.11	1.94 ± 0.09	0.1823
**Females**				
	**CON**	**FS**	**FC**	**P-Value**
Hb (mg/L)	18.1 ± 1.9	17.1 ± 4.3	17.0 ± 1.5	0.6475
Fe (ppm)				
Liver	172.8 ± 36.1	144.2 ± 29.9	169.5 ± 25.9	0.1536
Spleen	284.4 ± 74.7	251.6 ± 105.5	258.9 ± 38.03	0.9567
Weight (g)				
Body	188.8 ± 15.0	194.2 ± 13.0	200.9 ± 12.3	0.0939
Liver	6.91 ± 0.47	7.44 ± 0.92	7.32 ± 0.83	0.1951
Brain	1.80 ± 0.06	1.78 ± 0.07	1.79 ± 0.06	0.5674

Supplementary Table S6. P-values from diversity statistical analyses in YA rats.

Alpha-Diversity Metric (Sampling Depth)	P-Value			
**Faith's PD[1]**	**Treatment[2]**	**FS vs. CON[3]**	**FC vs. CON[3]**	FC vs. FS[3]
(1)	0.6783	0.1953	0.4034	0.2814
(1112)	<0.0001	0.0129	0.0022	<0.0001
(2223)	<0.0001	0.0103	0.0039	<0.0001
(3334)	<0.0001	0.0078	0.0047	<0.0001
(4445)	<0.0001	0.0016	0.0052	<0.0001
(5556)	<0.0001	0.0019	0.0052	<0.0001
(6667)	<0.0001	0.0018	0.0053	<0.0001
(7778)	<0.0001	0.0006	<0.0001	0.0144
(8889)	<0.0001	0.0011	0.0090	<0.0001
(10,000)	<0.0001	0.0015	0.0086	<0.0001
Shannon[4]	Treatment[2]	FS vs. CON[3]	FC vs. CON[3]	FC vs. FS[3]
(1)	NA			
(1112)	<0.0001	0.0005	0.0115	<0.0001
(2223)	<0.0001	0.0007	0.0143	<0.0001
(3334)	<0.0001	0.0009	0.0117	<0.0001
(4445)	<0.0001	0.0004	0.0150	<0.0001
(5556)	<0.0001	0.0004	0.0152	<0.0001
(6667)	<0.0001	0.0004	0.0150	<0.0001
(7778)	<0.0001	0.0004	0.0168	<0.0001
(8889)	<0.0001	0.0004	0.0184	<0.0001
(10,000)	<0.0001	0.0005	0.0176	<0.0001
ASVs[5]	Treatment[2]	FS vs. CON[3]	FC vs. CON[3]	FC vs. FS[3]
(1)	NA			
(1112)	<0.0001	0.0009	0.0139	<0.0001
(2223)	<0.0001	0.0006	0.0236	<0.0001
(3334)	<0.0001	0.0010	0.0189	<0.0001
(4445)	<0.0001	0.0003	0.0180	<0.0001
(5556)	<0.0001	0.0003	0.0173	0.0001
(6667)	<0.0001	0.0003	0.0166	<0.0001
(7778)	<0.0001	0.0003	0.0169	<0.0001
(8889)	<0.0001	0.0002	0.0278	<0.0001
(10,000)	<0.0001	0.0002	0.0267	<0.0001

	**P-Value[6]**				
**Beta-Diversity Metric**	Treatment	Sex	Treatment: Litter	Treatment: Sex	Treatment: Sex:Litter
Weighted UniFrac	0.0034	0.9558	0.4016	0.6150	0.8102
Unweighted UniFrac	<0.0001	0.1021	<0.0001	0.2973	0.2322
Bray-Curtis	<0.0001	0.0644	<0.0001	0.2727	0.0244

[1] Faith's Phylogenetic Diversity. [2] Kruskal-Wallis test for treatment effect. [3] Dunn's pairwise group comparisons. [4] Shannon's Diversity. [5] Amplicon Sequence Variant count (richness). [6] Permutational analysis of variance (PERMANOVA)

Supplementary Table S7. Young adult rat Phylum differential abundance supplementation group comparisons.

Phylum	Comparison	baseMean	log2FoldChange	padj
Actinobacteria	FS_vs_CON	521.634916	-0.0076584	0.98918101
Bacteroidetes	FS_vs_CON	1092.95573	0.46734273	0.56773788
Cyanobacteria	FS_vs_CON	4.77673283	0.6021831	0.4807081
Deferribacteres	FS_vs_CON	52.9791407	-0.8085905	0.21032867
Firmicutes	FS_vs_CON	37104.3337	-0.2605321	0.52908704
Patescibacteria	FS_vs_CON	3.82646994	-1.1051869	0.21032867
Proteobacteria	FS_vs_CON	262.734388	-3.9526979	3.10E-14
Tenericutes	FS_vs_CON	52.8866125	0.04124699	0.98918101
Verrucomicrobia	FS_vs_CON	394.528763	0.05637982	0.98918101
Unknown	FS_vs_CON	4.39389495	-2.2072996	0.00199727
Actinobacteria	FC_vs_CON	521.634916	-3.185122	4.10E-07
Bacteroidetes	FC_vs_CON	1092.95573	-5.3154188	7.81E-18
Cyanobacteria	FC_vs_CON	4.77673283	1.95798226	0.00101018
Deferribacteres	FC_vs_CON	52.9791407	0.8934887	0.08971613
Firmicutes	FC_vs_CON	37104.3337	0.06089323	0.82395484
Patescibacteria	FC_vs_CON	3.82646994	1.51685881	0.02208081
Proteobacteria	FC_vs_CON	262.734388	-3.6236625	7.58E-11
Tenericutes	FC_vs_CON	52.8866125	-1.6483561	0.00101018
Verrucomicrobia	FC_vs_CON	394.528763	1.74684294	0.00126733
Unknown	FC_vs_CON	4.39389495	0.22675945	0.81159844
Actinobacteria	FC_vs_FS	521.634916	-3.1774636	4.97E-07
Bacteroidetes	FC_vs_FS	1092.95573	-5.7827616	2.16E-21
Cyanobacteria	FC_vs_FS	4.77673283	1.35579916	0.01203638
Deferribacteres	FC_vs_FS	52.9791407	1.70207924	0.00092951
Firmicutes	FC_vs_FS	37104.3337	0.32142529	0.26169019
Patescibacteria	FC_vs_FS	3.82646994	2.6220457	8.07E-05
Proteobacteria	FC_vs_FS	262.734388	0.32903538	0.53824442
Tenericutes	FC_vs_FS	52.8866125	-1.6896031	0.00064748
Verrucomicrobia	FC_vs_FS	394.528763	1.69046312	0.00142814
Unknown	FC_vs_FS	4.39389495	2.43405904	0.00064748

Supplementary Table S8. Young adult rat Genus differential abundance supplementation group comparisons.

Genus	Comparison	baseMean	log2FoldChange	padj
Lactobacillus	FS_vs_CON	2483.7457	-13.03162978	1.91E-55
Lactobacillus	FC_vs_CON	2483.7457	-11.9770344	1.54E-41
Turicibacter	FS_vs_CON	148.167448	-8.983425394	8.14E-35
Turicibacter	FC_vs_CON	148.167448	-7.923251714	4.69E-24
uncultured bacterium	FC_vs_FS	818.083013	-7.521156478	3.29E-30
Ruminiclostridium 9	FC_vs_FS	218.961501	-6.922327075	8.82E-28
uncultured bacterium	FC_vs_CON	818.083013	-6.905999698	3.38E-25
Enterorhabdus	FC_vs_FS	99.8269249	-6.887098457	2.36E-26
[Eubacterium] ruminantium group	FC_vs_FS	89.6268716	-6.603413348	2.39E-11
Bacteroides	FC_vs_FS	229.64174	-6.44506143	2.32E-23
A2	FC_vs_FS	101.391184	-6.333072906	1.13E-21
GCA−900066575	FC_vs_FS	255.51557	-6.327545592	2.63E-22
Butyricicoccus	FC_vs_FS	89.1224262	-6.318895306	1.13E-21
Lachnospiraceae UCG−001	FC_vs_FS	1380.3985	-6.134109613	9.48E-17
Lachnospiraceae FCS020 group	FC_vs_FS	175.855168	-6.111987018	3.26E-22
Prevotellaceae NK3B31 group	FC_vs_FS	88.9237736	-6.074822995	1.13E-21
Dorea	FC_vs_FS	59.3152826	-6.06387171	1.16E-22
[Eubacterium] xylanophilum group	FC_vs_FS	1503.59081	-6.043506036	5.61E-23
Butyricicoccus	FC_vs_CON	89.1224262	-6.033874434	4.99E-19
Lachnospiraceae UCG−001	FC_vs_CON	1380.3985	-6.005018324	1.88E-15
Lachnoclostridium	FC_vs_FS	941.055958	-5.825335273	2.94E-20
Parabacteroides	FC_vs_FS	136.702842	-5.817975789	2.47E-24
Acetatifactor	FC_vs_FS	105.120581	-5.8052612	2.07E-19
Prevotellaceae UCG−001	FC_vs_FS	249.412684	-5.799364282	2.91E-20
Acetitomaculum	FC_vs_FS	144.340698	-5.62015411	7.83E-21
[Eubacterium] ventriosum group	FC_vs_FS	52.2457487	-5.585730775	5.07E-17
Ruminiclostridium 9	FC_vs_CON	218.961501	-5.533820778	1.01E-17
Roseburia	FC_vs_FS	2396.83108	-5.499657081	1.04E-18
Bacteroides	FC_vs_CON	229.64174	-5.493030086	7.97E-17
Lachnospiraceae UCG−006	FC_vs_FS	412.586107	-5.489149124	3.55E-18
Anaerostipes	FC_vs_FS	294.274519	-5.474644475	2.28E-10
Ruminococcaceae NK4A214 group	FC_vs_FS	35.1980214	-5.459692383	3.63E-21
Enterorhabdus	FC_vs_CON	99.8269249	-5.454920188	1.24E-16
[Eubacterium] ruminantium group	FC_vs_CON	89.6268716	-5.378929193	1.15E-07
uncultured Barnesiella sp.	FC_vs_CON	226.242545	-5.227605556	1.17E-07
[Eubacterium] ventriosum group	FC_vs_CON	52.2457487	-5.197475841	2.29E-14
Acetitomaculum	FC_vs_CON	144.340698	-5.176374216	4.56E-17
Roseburia	FC_vs_CON	2396.83108	-5.143050954	8.04E-16
uncultured bacterium	FC_vs_FS	29.5323043	-5.075861289	9.21E-16
A2	FC_vs_CON	101.391184	-4.982591621	1.92E-13
uncultured Barnesiella sp.	FC_vs_FS	226.242545	-4.918731916	4.09E-07
Dorea	FC_vs_CON	59.3152826	-4.90685174	7.49E-15
Bifidobacterium	FC_vs_CON	654.431753	-4.904175571	6.06E-11
Blautia	FC_vs_FS	390.195135	-4.898503227	4.64E-14
Bifidobacterium	FC_vs_FS	654.431753	-4.880602147	3.71E-11
Lachnospiraceae FCS020 group	FC_vs_CON	175.855168	-4.856665745	4.62E-14
Blautia	FC_vs_CON	390.195135	-4.797562981	4.20E-13
[Eubacterium] xylanophilum group	FC_vs_CON	1503.59081	-4.702148123	4.62E-14
Prevotellaceae UCG−001	FC_vs_CON	249.412684	-4.61914502	7.45E-13
Lachnospiraceae NK4B4 group	FC_vs_FS	19.9719551	-4.618470482	2.00E-09
Coriobacteriaceae UCG−002	FC_vs_FS	102.389941	-4.615111992	1.02E-10
GCA−900066575	FC_vs_CON	255.51557	-4.573171101	5.80E-12

Prevotellaceae NK3B31 group	FC_vs_CON	88.9237736	-4.552936459	2.50E-12
Alistipes	FC_vs_FS	24.4990952	-4.544697067	5.36E-12
Lachnoclostridium	FC_vs_CON	941.055958	-4.530249463	2.51E-12
uncultured	FC_vs_FS	228.108237	-4.498742077	2.82E-13
Ruminococcaceae UCG−004	FC_vs_FS	15.0495545	-4.487837851	2.05E-11
Adlercreutzia	FC_vs_FS	18.9445377	-4.220372348	1.93E-09
Anaerostipes	FC_vs_CON	294.274519	-4.197554403	2.57E-06
Lachnospiraceae UCG−006	FC_vs_CON	412.586107	-4.172077506	1.25E-10
Parabacteroides	FC_vs_CON	136.702842	-4.133868635	1.14E-12
Lachnospiraceae UCG−004	FC_vs_FS	13.1743277	-4.048776446	6.79E-10
Alistipes	FC_vs_CON	24.4990952	-4.005588127	2.58E-09
Acetatifactor	FC_vs_CON	105.120581	-3.925544886	3.50E-09
Escherichia−Shigella	FS_vs_CON	196.323115	-3.910953104	6.08E-11
uncultured Bacteroidales bacterium	FC_vs_CON	16.9449094	-3.869405751	1.52E-07
Tyzzerella 4	FS_vs_CON	4.8313218	-3.857112228	5.90E-11
uncultured	FC_vs_FS	1970.97886	-3.855610183	8.22E-11
uncultured bacterium	FC_vs_CON	29.5323043	-3.798875465	5.11E-09
Ruminococcaceae NK4A214 group	FC_vs_CON	35.1980214	-3.770428401	2.37E-10
Escherichia−Shigella	FC_vs_CON	196.323115	-3.767309695	8.53E-10
Intestinimonas	FC_vs_FS	13.9657251	-3.766726351	5.00E-08
Parasutterella	FC_vs_FS	15.6970085	-3.687651541	2.45E-06
uncultured Bacteroidales bacterium	FC_vs_FS	16.9449094	-3.60834736	7.08E-07
Parasutterella	FC_vs_CON	15.6970085	-3.536437014	1.02E-05
Adlercreutzia	FC_vs_CON	18.9445377	-3.501672362	1.32E-06
Coriobacteriaceae UCG−002	FC_vs_CON	102.389941	-3.266877947	1.02E-05
Tyzzerella 3	FC_vs_FS	52.7342782	-3.21496406	1.12E-06
GCA-900066225	FC_vs_CON	13.1496662	-3.159769794	2.95E-08
uncultured bacterium	FC_vs_CON	8.28627775	-3.133161259	7.27E-06
Intestinimonas	FC_vs_CON	13.9657251	-3.070547053	1.62E-05
Ruminococcaceae UCG−009	FC_vs_FS	27.3004252	-3.046940479	1.07E-06
Staphylococcus	FS_vs_CON	3.90070357	-2.973515836	1.92E-07
Enterococcus	FS_vs_CON	5.68524503	-2.876835461	5.98E-06
uncultured	FC_vs_CON	228.108237	-2.804476839	1.21E-05
Tyzzerella 4	FC_vs_CON	4.8313218	-2.776601587	7.91E-06
Tyzzerella 3	FC_vs_CON	52.7342782	-2.775846732	4.39E-05
Unknown	FC_vs_FS	4288.50063	-2.730573335	2.19E-07
Lachnospiraceae NK4B4 group	FC_vs_CON	19.9719551	-2.698180066	0.00097877
uncultured Firmicutes bacterium	FC_vs_FS	5.80591515	-2.682566894	2.77E-05
uncultured	FC_vs_CON	1970.97886	-2.626968519	1.94E-05
[Eubacterium] fissicatena group	FS_vs_CON	5.11710993	-2.62402099	3.07E-05
Acinetobacter	FS_vs_CON	2.51676391	-2.599637331	5.58E-06
Coprococcus 2	FS_vs_CON	2.27840207	-2.481723318	3.07E-05
Clostridiales bacterium enrichment culture clone 06−1235251−67	FC_vs_FS	6.87410323	-2.446598154	2.77E-05
Anaerotruncus	FC_vs_CON	28.0065901	-2.053291983	0.01017686
uncultured Lachnospiraceae bacterium	FC_vs_CON	4.81583123	-2.036730851	0.00315994
Lachnospiraceae UCG−004	FC_vs_CON	13.1743277	-2.036251602	0.00451245
uncultured bacterium	FC_vs_FS	8.28627775	-2.035791734	0.00468675
GCA−900066225	FS_vs_CON	13.1496662	-1.961953158	0.00038257
uncultured Mollicutes bacterium	FC_vs_FS	4.62196605	-1.929490726	0.00846291
uncultured rumen bacterium	FC_vs_FS	3.79758732	-1.856757316	0.00523879
Unknown	FC_vs_CON	4288.50063	-1.822956469	0.00097877
uncultured	FC_vs_FS	3.8856885	-1.794060745	6.59E-05
Phocea	FS_vs_CON	1.80222932	-1.731540442	0.00189098
uncultured Lachnospiraceae bacterium	FC_vs_FS	4.81583123	-1.635921521	0.01855382

Ruminococcaceae UCG−009	FC_vs_CON	27.3004252	-1.63159425	0.01714721
Acinetobacter	FC_vs_CON	2.51676391	-1.517621202	0.01309167
uncultured bacterium	FS_vs_CON	1.78925779	-1.481726819	0.01287161
Coprococcus 2	FC_vs_CON	2.27840207	-1.400436422	0.03022422
Ruminiclostridium 5	FC_vs_FS	271.524596	-1.310604025	0.00729618
GCA−900066225	FC_vs_FS	13.1496662	-1.197816636	0.04814768
Streptococcus	FS_vs_CON	1.84340814	-1.183844324	0.02908293
uncultured	FC_vs_FS	2.41233768	-1.142820552	0.0290884
Ruminococcaceae UCG−014	FC_vs_FS	674.18926	-1.126082222	0.03741428
uncultured	FC_vs_FS	4.60712959	0.980960401	0.03741428
Anaerofustis	FC_vs_FS	1.44894849	1.102563912	0.04285036
Bacillus	FC_vs_FS	1.39188411	1.105422455	0.03946832
Candidatus Saccharimonas	FC_vs_FS	1.35583732	1.107274449	0.03765589
Anaerofilum	FC_vs_FS	1.34804059	1.107450254	0.03765589
Dietzia	FC_vs_FS	1.30816026	1.108240165	0.03741428
Clostridium sensu stricto 18	FC_vs_FS	1.30816026	1.108240165	0.03741428
uncultured organism	FC_vs_FS	1.30816026	1.108240165	0.03741428
[Ruminococcus] gauvreauii group	FC_vs_FS	1.30816026	1.108240165	0.03741428
Lachnoclostridium 5	FC_vs_FS	1.30816026	1.108240165	0.03741428
Lachnospiraceae NC2004 group	FC_vs_FS	1.30816026	1.108240165	0.03741428
Moryella	FC_vs_FS	1.30816026	1.108240165	0.03741428
Sellimonas	FC_vs_FS	1.30816026	1.108240165	0.03741428
Candidatus Soleaferrea	FC_vs_FS	1.30816026	1.108240165	0.03741428
Haemophilus	FC_vs_FS	1.30816026	1.108240165	0.03741428
Defluviitaleaceae UCG−011	FC_vs_FS	25.9337856	1.121021492	0.02225898
uncultured rumen bacterium	FC_vs_CON	1.46654416	1.126332484	0.03283721
Ruminococcaceae UCG−005	FC_vs_FS	245.305711	1.154586719	0.03741428
Ruminococcus 1	FC_vs_CON	1421.96818	1.170878701	0.04506375
Oscillibacter	FC_vs_CON	591.22098	1.176264449	0.03405798
uncultured bacterium	FS_vs_CON	2.72067845	1.183692057	0.04990854
Tyzzerella	FC_vs_CON	87.3770525	1.244725504	0.02395119
uncultured	FS_vs_CON	2.41233768	1.245642067	0.01587785
uncultured bacterium	FS_vs_CON	29.5323043	1.276985824	0.04990854
unidentified rumen bacterium RF32	FC_vs_FS	2.98292233	1.318179504	0.00478197
Defluviitaleaceae UCG−011	FC_vs_CON	25.9337856	1.324107749	0.00750157
uncultured	FC_vs_CON	4.60712959	1.372995516	0.0052082
Hydrogenoanaerobacterium	FC_vs_FS	1.38027696	1.373524469	0.00712283
Facklamia	FC_vs_FS	1.71257229	1.383734529	0.00527655
Ruminiclostridium 9	FS_vs_CON	218.961501	1.388506297	0.04990854
Ruminococcaceae UCG−009	FS_vs_CON	27.3004252	1.415346229	0.04990854
Enterorhabdus	FS_vs_CON	99.8269249	1.432178269	0.02900559
Family XIII AD3011 group	FC_vs_FS	9.75065878	1.43810261	0.00844407
Rothia	FS_vs_CON	10.1242213	1.446722049	0.00814556
Fournierella	FS_vs_CON	10.8224695	1.451646592	0.04824195
Family XIII AD3011 group	FC_vs_CON	9.75065878	1.453160392	0.0101334
uncultured	FC_vs_FS	1.41146118	1.483150942	0.00318402
uncultured Firmicutes bacterium	FS_vs_CON	5.80591515	1.512693325	0.0196532
Prevotellaceae NK3B31 group	FS_vs_CON	88.9237736	1.521886536	0.02669235
[Eubacterium] nodatum group	FC_vs_FS	40.2564595	1.572278329	0.00070517
uncultured rumen bacterium	FC_vs_FS	1.46654416	1.638420354	0.00075047
Parabacteroides	FS_vs_CON	136.702842	1.684107154	0.0064322
Ruminococcaceae NK4A214 group	FS_vs_CON	35.1980214	1.689263982	0.00153038
uncultured	FS_vs_CON	228.108237	1.694265238	0.01781735
Rothia	FC_vs_CON	10.1242213	1.737132396	0.00061171
GCA−900066575	FS_vs_CON	255.51557	1.75437449	0.01781735

[Eubacterium] coprostanoligenes group	FC_vs_FS	566.387421	1.793344316	0.0033409
Ruminiclostridium 1	FC_vs_CON	4.17427591	1.874776632	0.00033156
unidentified rumen bacterium RF32	FC_vs_CON	2.98292233	1.878159876	0.00017847
Acetatifactor	FS_vs_CON	105.120581	1.879716314	0.00769312
Lachnospiraceae NK4B4 group	FS_vs_CON	19.9719551	1.920290416	0.01788363
Caldicoprobacter	FC_vs_CON	3.93015968	1.923971143	0.00046188
Brevibacterium	FC_vs_CON	1.89614918	1.931591296	8.63E-05
Papillibacter	FC_vs_CON	4.79006649	1.976294183	0.00108651
Faecalibaculum	FC_vs_FS	353.034549	1.984640813	0.01738563
Lachnospiraceae UCG−004	FS_vs_CON	13.1743277	2.012524844	0.00157282
uncultured organism	FC_vs_CON	2.19470567	2.102292434	4.39E-05
Corynebacterium 1	FC_vs_FS	6.08686737	2.351108445	0.00011202
Enterococcus	FC_vs_FS	5.68524503	2.397013259	0.00017567
Brevibacterium	FC_vs_FS	1.89614918	2.427562279	2.89E-07
Yaniella	FC_vs_CON	2.40554171	2.448047862	7.76E-06
[Eubacterium] fissicatena group	FC_vs_FS	5.11710993	2.468846855	7.07E-05
Ruminococcus 2	FC_vs_CON	318.955147	2.479216544	0.00797728
Caldicoprobacter	FC_vs_FS	3.93015968	2.501776208	2.44E-06
Ruminiclostridium 1	FC_vs_FS	4.17427591	2.597630494	3.40E-07
uncultured organism	FC_vs_FS	2.19470567	2.649339441	1.21E-07
uncultured	FS_vs_CON	3.8856885	2.664411264	4.25E-09
Staphylococcus	FC_vs_FS	3.90070357	2.68159796	3.36E-06
Brachybacterium	FC_vs_CON	2.76649668	2.715679448	1.06E-06
Papillibacter	FC_vs_FS	4.79006649	2.838054631	1.39E-06
Yaniella	FC_vs_FS	2.40554171	2.921531599	3.16E-08
Brachybacterium	FC_vs_FS	2.76649668	3.184846508	3.02E-09
